# Alzheimer’s disease brain-derived tau extracts show differential processing and transcriptional effects in human astrocytes

**DOI:** 10.1016/j.isci.2025.112793

**Published:** 2025-05-30

**Authors:** Matthew J. Reid, Melissa Leija Salazar, Claire Troakes, Steven Lynham, Deepak P. Srivastava, Beatriz Gomez Perez-Nievas, Wendy Noble

**Affiliations:** 1 King’s College London, Institute of Psychiatry, Psychology and Neuroscience, Department of Basic and Clinical Neuroscience, 5 Cutcombe Road, London SE5 9RX, UK; 2 University College London, Institute of Neurology, Rowland Hill Street, London NW3 5NJ, UK; 3 London Neurodegenerative Diseases Brain Bank, Institute of Psychiatry, Psychology and Neuroscience, Kings College London, London, UK; 4 King’s College London, Proteomics Core Facility, James Black Centre, London SE5 9NU, UK; 5 University of Exeter, Department of Clinical and Biomedical Sciences, Prince of Wales Road, Exeter EX4 4PS, UK

**Keywords:** Cell biology, Cellular neuroscience, Neuroscience, Omics

## Abstract

Post-translational modifications of tau, including phosphorylation at specific residues, are closely linked with tau seeding ability and clinical disease progression. While most previous evidence has focused on neuronal tau spread, evidence supports a similar role for astrocytes. Here, we demonstrate that well characterized tau aggregates isolated from postmortem Alzheimer’s disease brain are internalized and processed by control human–induced pluripotent stem cell-derived astrocytes. Differences in the efficiency of tau internalization, clearance and/or seeding were noted, which reflect molecular properties of tau and/or co-factors in brain extracts. We observed a direct relationship between tau handling by astrocytes and astrocyte transcriptomic changes. Dysregulated genes include several previously identified as upregulated in reactive astrocytes in Alzheimer’s brain, as well as those implicated in pathological tau clearance by autophagy and other pathways. The study provides insights into the complex interplay between tau molecular diversity and astrocyte responses in Alzheimer’s disease.

## Highlights

• Human iPSC-derived astrocytes express efficiently internalize tau aggregates
• The internalized tau can be cleared from astrocytes or seed further aggregation
• Tau handling profiles are linked to molecular properties of tau or the extracts
• Differences in tau handling are reflected in astrocyte transcriptional changes

## Graphical abstract

**Figure:**
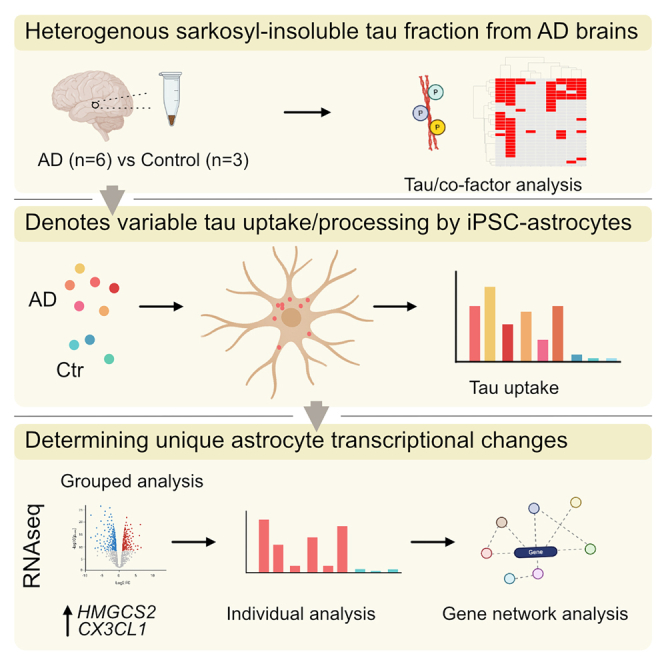

## Introduction

Altered interactions between neurons and glia contribute to the development and progression of many neurodegenerative diseases, including Alzheimer’s disease (AD), in which the prion-like spread of protein aggregates accompanies disease worsening. Glial cell function is altered in response to Aβ and tau aggregates,^1^^,^^2^ and understanding the consequences of these alterations may be key to determining the cellular mechanisms that drive the spread of pathological forms of tau through affected brain regions.

Tau forms the main component of neurofibrillary tangles in AD, as well as distinct neuropathological hallmarks in other tauopathies.^3^ The extensive modifications of tau in AD brain are now largely well understood,^4^ as are the structure of tau aggregates in AD brain.^5^^,^^6^ However, there is considerable variation in the molecular profile of tau between individuals, with modifications such as phosphorylation at specific residues being associated with tau seeding efficiency and clinical disease progression.^7^ This reflects evidence that several tau “strains” exist in each tauopathy, with variation both within and between tauopathy groups.^8^ Whether and how molecular diversity of tau affects glia in AD is not fully understood.

Astrocytes are the most abundant cell type of the brain,^9^ providing homeostatic support for neurons and helping to regulate synaptic activity^10^^,^^11^ among other functions. In AD, heterogeneous astrocyte responses are determined by spatiotemporal stage.^12^ Astrocytes associate with neurofibrillary tangles as well as Aβ plaques,^13^ and harbor tau inclusions in the dentate gyrus^14^ which may highlight a potential role of these cells in tau spread. Indeed, astrocytes accumulate pathological tau following its spread from neurons in mice.^15^ Astrocyte phagocytic ability^16^ allows them to indirectly internalize large tau fibrils along with dead or dying neurons,^17^ and while the ability of astrocytes to directly internalize synthetic tau fibrils has also been demonstrated,^18^^,^^19^ their conformational structure is now understood to be different from that of AD and other tauopathies.^20^

The aim of this study was to determine if the molecular properties of AD brain–derived tau influence the rate of human tau aggregate uptake by human astrocytes and/or affect the function of astrocytes. We found tau was internalized by astrocytes, but that the rate of uptake and clearance varied for tau isolated from different AD cases. The molecular properties of tau, as defined through analysis of post-translational modifications, showed associations with both the rate of tau uptake and either seeding of endogenous tau or clearance by human astrocytes, as well as changes in astrocyte function indicated by gene expression changes. Other components of brain extracts may also influence these events. These data suggest that astrocytes also make an important contribution to the rate of tau spread in AD, which may be further confounded by changes in astrocyte function.

## Results

### Tau accumulates in astrocytes in AD temporal cortex

**Figure 1:**
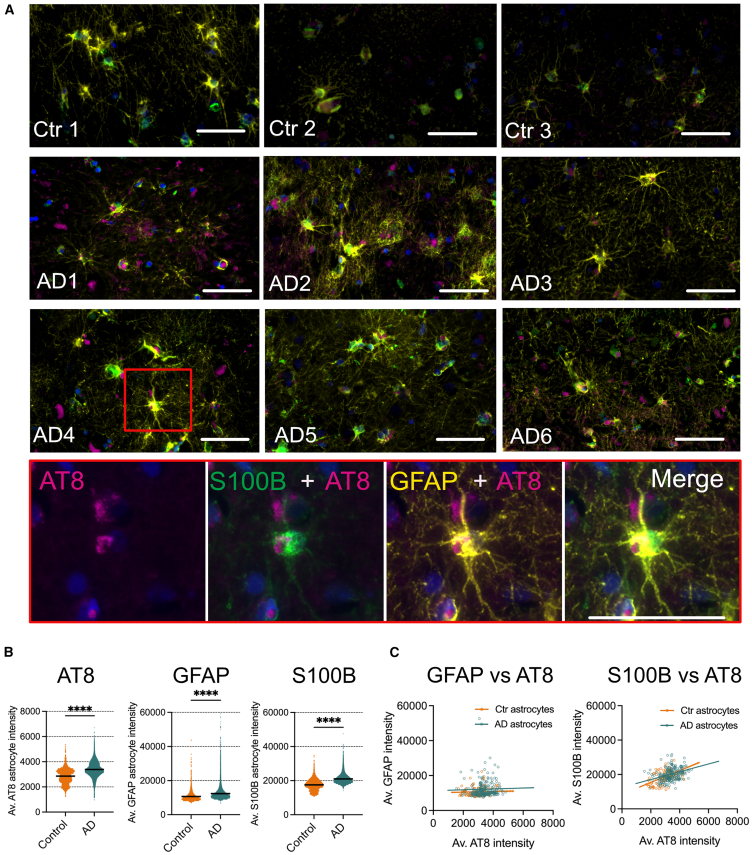
Characterization of human postmortem temporal cortex shows tau inclusions associated with astrocytes (A) Representative immunolabeling of astrocytes with antibodies against GFAP (yellow), S100B (green) and AT8 (purple) in temporal cortex tissue sections from AD 6 cases and 3 control brains. Lower panel shows higher magnification image of area indicated by the red box in AD4. Scale bar = 50 μm. (B) Scatterplots of AT8, GFAP and S100B intensity in individual astrocytes from AD (*n* = 6) and Control (*n* = 3) brain sections. Black lines represent mean intensities. (C) Pearson correlation analysis of intensity of AT8 immunolabeling relative to GFAP or S100B in individual astrocytes in temporal cortex of AD (*n* = 6) and control sections (*n* = 3). Data in (B) and (C) were analyzed from cells positive for both GFAP and S100B in gray matter of temporal cortex, for a combined total of 68510 astrocytes in AD tissue sections and 3217 astrocytes in control tissue sections using an unpaired t-test in (A) and Pearson correlation analysis in (B), ∗∗∗∗*p* < 0.0001.

Although the accumulation of disease-associated forms of tau in astrocytes is a characteristic feature of several primary tauopathies, it is not commonly described in AD.^21^^,^^22^ However, tau accumulation within hilar astrocytes of the dentate gyrus in AD brain was recently shown to mediate neuronal dysfunction and cognitive decline.^14^ We examined the association of AT8+ve tau with astrocytes in Braak stage V-VI AD brain (*n* = 6) relative to control (non-neurologically impaired, (*n* = 3)) temporal cortex (BA21) gray matter. AD tissues showed the expected accumulation of sarkosyl-insoluble tau aggregates when examined biochemically (Figure S1A). AT8+ tau structures were commonly detected within AD tissue, and to a lesser extent in control tissues (Figure 1A), and while most showed little colocalization with astrocytes, some AT8+ structures in AD sections were identified in glial fibrillary acidic protein (GFAP) and S100 calcium binding protein-b (S100b)-labeled astrocytes (Figure 1A). The average AT8 levels in AD astrocytes were significantly higher than in control cases. Although there was significant variation between individual cases (F (8, 71722) = 5229, *p* < 0.0001, R^2^ = 0.3684) (Figure S1B), these data suggest that some disease-modified tau is present in astrocytes in AD brain.

Astrocytes in AD brain are typically characterized by high levels of GFAP,^13^ as found here (Figure 1B). S100b is a calcium binding protein expressed by astrocytes that was recently shown to hinder tau aggregation and seeding.^23^ S100b immunoreactivity (Figure 1B) was significantly higher in AD tissues relative to controls (F (8, 71722) = 5096, *p* < 0.0001, R^2^ = 0.3624), as with GFAP (F (8, 71723) = 232.7, *p* < 0.0001, R^2^ = 0.02530), with significant variation between cases (Figure S1B).

Pearson correlation analysis showed a neutral correlation between AT8 and GFAP intensity in both AD (r = 0.032) and Ctr samples (r = 0.035), whereas there was a positive correlation between AT8 and S100B for both Ctr (r = 0.554) and AD (r = 0.338) (Figure 1C). Again, there was variation between individual cases, with correlation between AT8 and S100B ranging from positive (Ctrl 1, Ctrl 3, AD1, AD4, and AD5) to none (Ctrl 2, AD6) (Figure S1C). Similarly, some cases showed weak positive correlation between GFAP and AT8 levels (Ctrl 3, AD1, AD2, and AD5), with others showing no correlation (Ctrl 1 and Ctrl 2) or weak negative correlation (AD3, AD4, and AD6) (Figures S1C and S1D). Collectively, these findings highlight a complex and variable astrocytic reaction to tau pathology in AD brain.

### Post-translational modifications of AD tau aggregates vary by case

**Figure 2:**
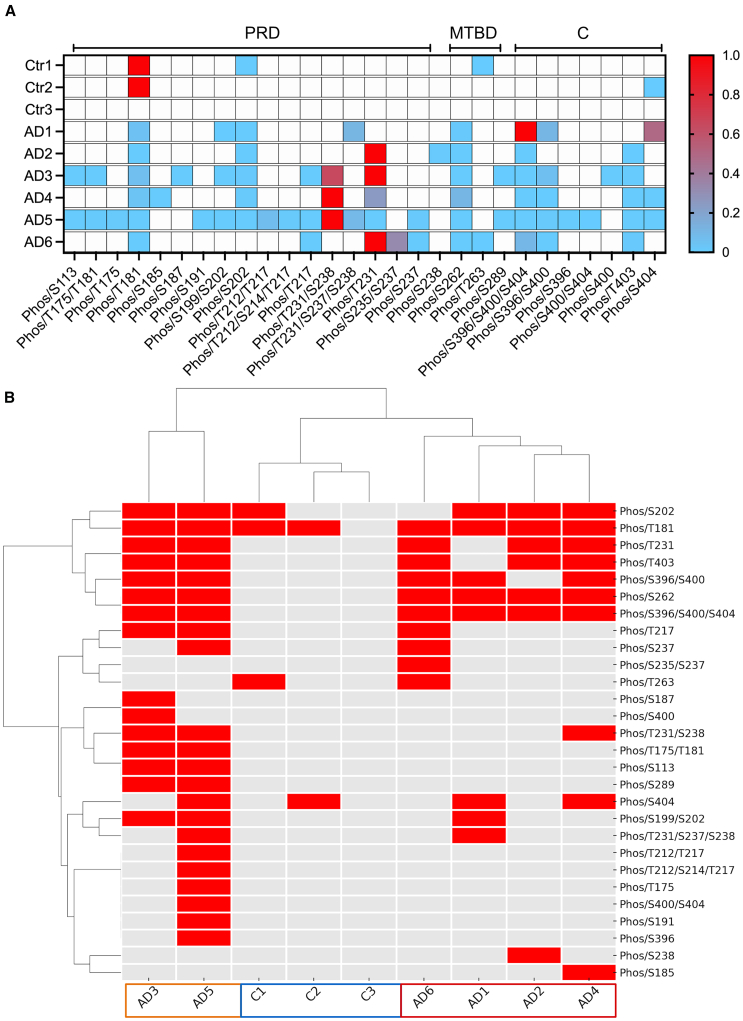
Characterization of sarkosyl-insoluble tau from postmortem human brain by LC-MS/MS (A) Heatmap of tau phosphorylation sites within individual AD and control brain samples, quantified as the ratio of modified (phosphorylated) to equivalent unmodified peptides and normalized between 0 (blue) and 1 (red) for each sample (gray = undetected) to show the relative abundance of tau phosphorylation sites within that sample. (B) The presence (red) or absence of phosphorylation at specific tau residues was used for unbiased hierarchical clustering of AD and control cases that determined 3 main groups: Ctr1-3; AD3,5; AD1,2,4,6. PRD, Proline rich domain; MTBD, microtubule binding domain; C, C-terminus.

Tau in AD brain shows several modifications according to disease stage,^4^ with significant variation in tau “strain”^8^ having consequences for tau seeding ability *in vitro,* as well as clinical outcomes.^7^^,^^24^ Here, liquid chromatography-tandem mass spectrometry (LC-TMS) was used to identify sites of tau modification in sarkosyl-insoluble fractions from the AD and control brain samples used in this study. Two control cases (Braak stage 0-I) showed a small number of phosphorylated residues predominantly in the proline-rich domain including threonine (T)181 and serine (S)202, which are associated with the early stages of tau aggregation,^4^ while any modifications present were below limits of detection for one control case. Phosphorylation at several residues were detected in the AD samples, particularly in the proline rich domain of tau, and to a lesser extent in the microtubule binding domain. All AD cases showed phosphorylation at some sites in the C-terminal domain (Figure 2A). The number of sites at which phosphorylation was detected and the extent of phosphorylation varied between AD case. A hierarchical cluster analysis based on the presence or absence of phosphorylation at specific sites yielded three groups. Controls 1–3, AD3 and AD5, and AD1, 2, 4, and 6 (Figure 2B). AD3 and AD5, which were the only cases phosphorylated at S113, T175, and S289, were placed in a distinct cluster from the other AD and control cases. Tau in AD6 did not show any phosphorylation between S185 and T217, whereas phosphorylation in this region was evident in the other five AD cases. All AD cases, but not controls, showed phosphorylation at S394/400/404 and S262, hallmark phosphorylation sites in AD^4^; S262 being linked with high tau seeding efficiency and disease worsening.^25^ It is important to note that low-abundance modifications may have been present in some samples, but these will not have met the stringent threshold for confident detection. This is a recognized limitation of MS-based PTM profiling, where high-confidence detection favors the most abundant modifications in a sample.

Peptide fragments from several other proteins were also detected in the sarkosyl-insoluble fraction (Figure S2). An unsupervised hierarchical cluster analysis of the most abundant proteins separated control from AD cases (Figure S2A). Within the AD cluster, AD3 and AD5 again appeared most distinct from AD1, AD2, AD4 and AD6 and the control cases. Proteins such as Trypsin-1, RNA-splicing ligase RtcB homolog, and Ubiquitin-40S ribosomal protein S27a were highest in AD3/5. These proteins are involved in proteolytic activity, RNA processing, and protein degradation, suggesting distinct molecular alterations in these AD cases. Combined, 55 proteins were detected that differed significantly between AD and control tissue (Figure S2B). The top most significantly enriched in AD brain included proteins involved in RNA processing such as U1 small nuclear ribonucleoprotein 70kDa (U1-70K), known to co-aggregate with AD tau^26^^,^^27^ and various small nuclear ribonucleoprotein-associated proteins, notable because RNA dysregulation may play a role in the progression of AD in some patients.^28^ Other proteins significantly upregulated in AD cases included commonly associated AD proteins such as tau, apolipoprotein E (APOE) and amyloid-beta precursor proteins. Several proteins associated with the complement system were also detected such as complement C3 and complement C4-A. Agrin, a heparan sulfate proteoglycan seen to accumulate in AD brain was also highly upregulated in these aggregated tau fractions.^29^ It is possible that some of these proteins may participate actively in disease development if co-factors contribute to tau aggregation, seeding and/or spread.

### Human astrocytes internalize AD brain derived tau aggregates at varying rates

Recent data show that human iPSC-derived astrocytes internalize neuronal debris^17^ and it might be reasoned that this accounts for tau immunolabeling in human PM brain astrocytes. However, growing evidence indicates that astrocytes can internalize different forms of human tau in an active process that might contribute to the spread of tau aggregates across diseased brain.^18^^,^^19^^,^^30^^,^^31^

Tau uptake and/or seeding is facilitated by the presence of similar forms of tau^32^^,^^33^ and single cell analysis of human brain shows that astrocytes express *MAPT*.^34^^,^^35^ Here, astrocytes were differentiated from control iPSCs using a well-characterized protocol.^36^ Seventy day after differentiation, these cells express robust levels of mature astrocyte genes (Figure S3A) and tau protein (Figures S3B–S3D). As specific tau isoforms, such as those expressed in mature brain, may be more prone to misfolding and recruitment to facilitate tau spread,^37^^,^^38^ we examined the expression of *MAPT* mRNA against alternatively spliced regions of tau using primer sequences that target exons 2 and 3 which encode the alternatively spliced N-terminal subunits of tau (0N, 1N, 2N tau) and exon 10 for the second microtubule binding domain (3R/4R) (Figure S3B). In iPSCs and NPCs, 0N and 3R tau isoforms were the predominant source of *MAPT* mRNA. NPCs showed higher total levels of *MAPT* expression compared to iPSCs. As NPCs were differentiated into astrocytes, the proportion of 1N and 4R *MAPT* mRNA increased, and relatively low levels of 2N *MAPT* mRNA emerged during astrocyte maturation. These data suggest that astrocytes derived from iPSCs recapitulate developmentally regulated tau splicing, as observed at a slower rate, in iPS-neurons.^39^

**Figure 3:**
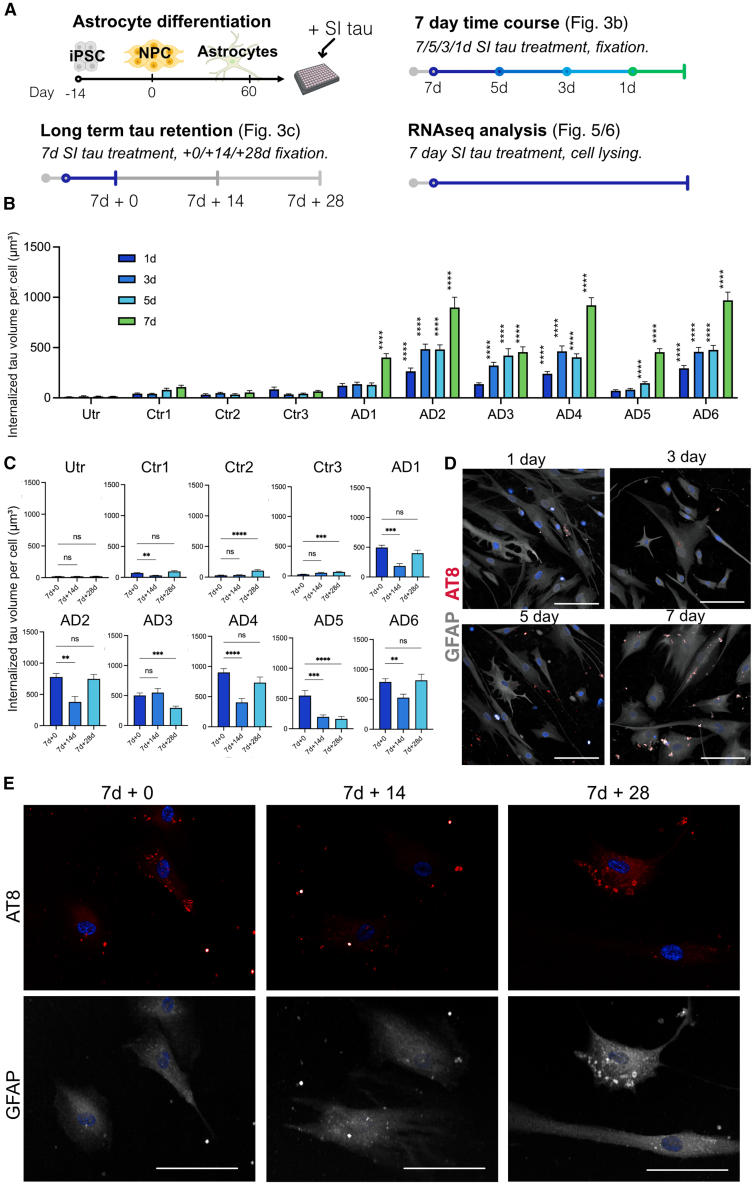
Astrocyte uptake of tau aggregates derived from AD postmortem tissue iPSC-astrocytes were incubated with 35 ng/mL of sarkosyl-insoluble tau derived from postmortem tissue of six AD cases and three equivalent control brain fractions. (A) Schematic diagram outlining the treatment of astrocytes differentiated from iPSC^36^ and treatment with sarkosyl-insoluble (SI) fractions from control and AD postmortem human brain for analysis of tau (AT8) content and to generate material for RNA-seq. (B) Average detected volume of internalized AT8 positive tau aggregates in astrocytes after 1, 3, 5, or 7 days of exposure to sarkosyl-insoluble tau fractions. (171–419 cells per treatment condition across 3 experiments, *n* = 3 control; *n* = AD 6). (C) Average detected volume of internalized AT8 tau after 7-day tau incubation (+0), and at +14 and +28 days after tau removal from media. (400–500 cells across 3 experiments, *n* = 3 control; *n* = AD 6). (D) Representative immunolabeling of AT8 positive tau (red) internalized within GFAP positive (gray) astrocytes at 1, 3, 5, and 7 days after exposure to AD1 tau. White scale bars = 100 μm. (E) Representative immunolabeling of AT8 positive tau (red) internalized within GFAP positive (gray) astrocytes after 7-day treatment (+0) and 14 days (7 days +14) or 28 days (7 days + 28) after tau removal. White scale bars = 100 μm. Data are from three independent differentiations of iPS-astrocytes. Data is mean ± SEM. Statistical analysis by two-way ANOVA with Tukey’s multiple comparisons test to untreated cells in (A) and one-way ANOVA with Dunnett’s multiple comparisons test to baseline (7 days + 0) in (B). ∗∗∗∗*p* < 0.0001, ∗∗∗*p* < 0.001, ∗∗*p* < 0.01, ∗*p* < 0.05, ns = not significant.

We next spiked the media of 70-day iPSC-derived astrocytes (Figure 3A) with tau aggregates from the AD and control cases, and tau uptake by astrocytes was measured over 7 days (Figure 3B). Immunolabeling of cells using the AT8 antibody allowed us to distinguish between endogenously expressed astrocytic tau (AT8 negative at baseline) and exogenously applied human tau (Figure 3D). Immunolabeling and 3D z stack imaging was used rather than western blotting since it provides critical spatial information that immunoblotting cannot capture, and 3D z-stacks allowed us to confirm that tau aggregates are truly internalized within astrocytes, rather than remaining attached to the cell surface or the culture plate. We found that astrocytes internalize tau in a time-dependent manner, showing greater volumes of AT8-positive tau aggregates after 7 days of incubation (Figure 3B). Although tau aggregates from each AD case were internalized, we observed varying rates of tau uptake depending on the AD case used, with the slowest rates of uptake observed with tau from cases AD1, AD3, and AD5. Next, we studied the longer-term dynamics of tau handling by astrocytes. Here, tau-containing medium was removed after 7 days and iPSC-astrocytes grown in standard media for a further 14 or 28 days. Analysis of AT8 content after 14 days showed reduced AT8 volume suggesting that astrocytes degraded or otherwise cleared tau aggregates following the removal of tau from culture media, with the exception of AD3 (Figure 3C). Examination of AT8 levels 28 days following tau removal from media showed increased AT8 reactivity suggesting that there was seeding of endogenous astrocyte tau in cases treated with tau aggregates from cases AD1, AD2, AD4, and AD6 (Figures 3C and 3E). This feature was not observed with tau from cases AD3 and AD5 which showed further reductions in AT8 intensity, in addition to unique phosphorylation events (pSer113, pThr175/181, and pS289) relative to the other samples.

In parallel experiments, the morphology of nuclei was examined as an indication of cell viability. A linear classification system that utilizes the pre-calculated size, intensity and roundness of Hoechst-stained nuclei was trained by machine learning to split cells into “healthy” and “dead or dying cells”.^40^ Analyses of these data showed no differences in astrocyte viability between groups or upon exposure to sarkosyl-insoluble brain extracts (Figure S4).

### Human tau aggregates sequester astrocytic GFAP and S100b

**Figure 4:**
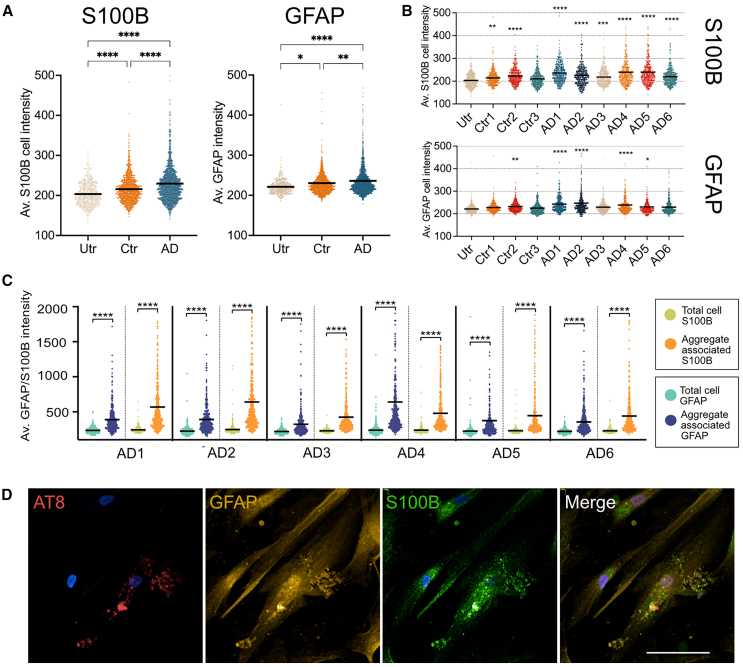
GFAP and S100B localize to internalized tau aggregates (A) Immunofluorescence intensity of GFAP and S100B in iPSC-astrocytes were measured after incubation with 35 ng/mL of sarkosyl-insoluble AD and control fractions for 7 days. (A) Scatterplots of average GFAP and S100B immunofluorescence intensity for combined AD treated (*n* = 6), control treated (*n* = 6) and untreated iPSC-astrocytes (2461, 1275, and 427 astrocytes respectively per treatment group across 3 experiments). (B) Scatterplots of average S100B and GFAP immunofluorescence intensity following exposure to tau from individual AD and control sarkosyl-insoluble fractions, relative to untreated iPSC-astrocytes (385–488 cells per treatment across 3 experiments, *n* = 3 Ctr, *n* = AD 6). (C) Scatterplots comparing mean “total cell intensity” relative to mean “aggregate-associated” tau intensity after internalization of AD sarkosyl-insoluble tau aggregates (*n* = 398–488 cells across 3 experiments, *n* = 3 Ctr, *n* = AD 6). (D) Representative immunolabeling showing GFAP (yellow) and S100B (green) localizing at high levels around internalized AT8-positive tau aggregates (red) in astrocytes exposed to sarkosyl-insoluble AD1 tau for 7 days. White scale bar = 50 μm. Data are from three independent differentiations of iPS-astrocytes. Black bar is mean of individual cell data. Statistical analysis by one-way ANOVA with Dunnett’s multiple comparisons test to untreated in (B), and paired t-test for GFAP/S100B total cell average vs. aggregate-associated immunofluorescence in (C). ∗∗∗∗*p* < 0.0001, ∗∗∗*p* < 0.001, ∗∗*p* < 0.01, ∗*p* < 0.05.

After 7 days of exposure to human AD brain tau aggregates, immunolabeling showed significantly increased average S100B immunoreactivity following exposure to sarkosyl-insoluble fractions, particularly from AD cases (Figure 4A). While variable, S100B was increased by exposure to all AD and control samples except for Ctr3 (Figure 4B). Similar effects were observed for GFAP (Figure 4A), where 4 of AD 6 cases showed significantly higher local GFAP immunoreactivity compared to only 1 of 3 control cases (Figure 4B). Pearson correlation analysis revealed significant associations between AT8 and both S100B and GFAP in astrocytes spiked with tau (Figure S5A). These correlations are significantly more robust than were found in the AD brain analysis, indicating that there are confounding factors other than tau uptake that can affect tau association with astrocytes in diseased human brain. However, we saw no significant global changes in *GFAP* or *S100B* gene expression relative to controls (Figure S5B).

There was a pronounced accumulation of both GFAP and S100B proximal to AT8+ tau within iPSC-astrocytes treated with tau from AD cases (Figures 4C and 4D) that persisted following tau removal from media (Figure S5C). These may suggest that GFAP and S100B are sequestered by tau inclusions, irrespective of whether seeding of endogenous tau was observed.

### Alterations to astrocyte gene expression upon tau aggregate internalization

**Figure 5:**
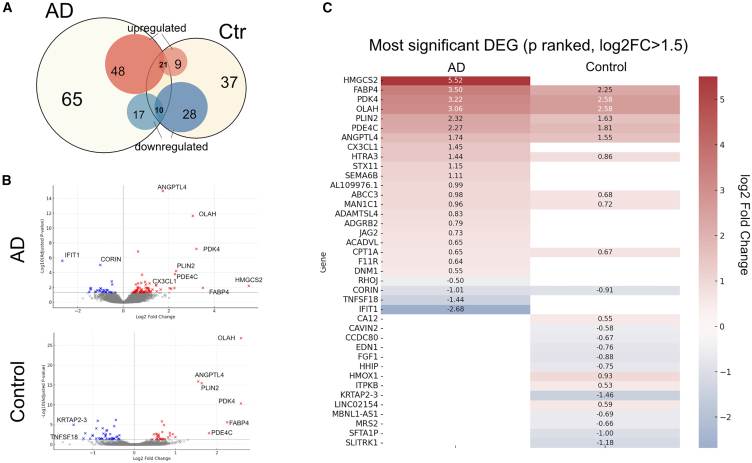
Differential gene expression in astrocytes after AD tau exposure iPSC-astrocytes were treated with 35 ng/mL of tau from sarkosyl-insoluble fraction of (AD1–6) and control (Ctr1-3) brains for 7 days and compared to untreated astrocytes after bulk RNA-sequencing. Data was pooled to compare AD treated (*n* = 6) and control treated (*n* = 3) gene expression changes to untreated astrocytes across 3 experimental repeats. (A) Venn diagram of significantly (*p* < 0.05) up (red) and down (blue) regulated genes in AD treated (left), control treated (right) and overlapping genes, relative to untreated astrocytes. (B) Volcano plot of significant upregulated (red) and downregulated (blue) DEGs (*p* < 0.05) in AD and control treated astrocytes vs. untreated controls, with annotation of the most significantly altered genes (*p* < 0.01). (C) Top DEGs in both control and AD groups ranked by lowest *p* values and filtered by log2FC > 1.5. Red gradient represents strength of upregulation and blue represents downregulation as per log2(Fold change) relative to untreated astrocytes.

Astrocytes become reactive in AD, showing significant alterations in their transcriptional regulation.^41^ To delineate broad gene expression changes common to the uptake of disease associated tau aggregates, bulk RNA-seq data from astrocytes treated with tau from AD cases or control brain extracts were grouped and compared to untreated astrocytes. Treatment with AD brain tau resulted in 96 significant (*p* < 0.05) differentially expressed genes (DEGs), while treatment with equivalent control brain fractions showed 68 DEGs, of which 31 genes were common to both AD and control treated astrocytes (Figures 5A and 5B). Of the overlapping genes, the correlation of fold change between both groups was very high (r = 0.992), indicating these genes are dysregulated in a similar manner. These likely reflect responses to other components of the sarkosyl-insoluble fractions used. Cells treated with control brain extracts showed a higher number of downregulated genes than was observed following spiking with AD tau, and the mean fold change for both up and downregulated genes was highest in the AD group.

The top 25 most significant DEGs (log2FC > 1.5) show several unique and significantly altered genes in astrocytes exposed to AD tau, with known roles in astrocyte signaling and the clearance of pathological protein aggregates. For example, chemokine *CX3CL1 (*2.73-fold upregulation; *p* < 0.01), regulates neuron-glial interactions in neurodegenerative diseases^42^^,^^43^ and is upregulated in astrocytes in disease (Lindia et al., 2005),^44^ *IFIT1* (interferon-induced protein with tetratricopeptide repeats 1) mediates astrocyte responses to viral infections^45^ and tumor necrosis factor (ligand) superfamily member 18 (*TNFSF18*) was downregulated 6.4-fold in the same treatment conditions. *HMGCS2* (mitochondrial 3-hydroxy-3-methylglutaryl-COA synthase 2), an enzyme that plays a key role in ketogenesis^46^ and is important for autophagic degradation of amyloid-beta precursor proteins^47^ and tau,^48^ showed a large (45.9-fold) upregulation, perhaps indicating this as a key pathway in astrocytic tau processing (Figure 5C).

**Figure 6:**
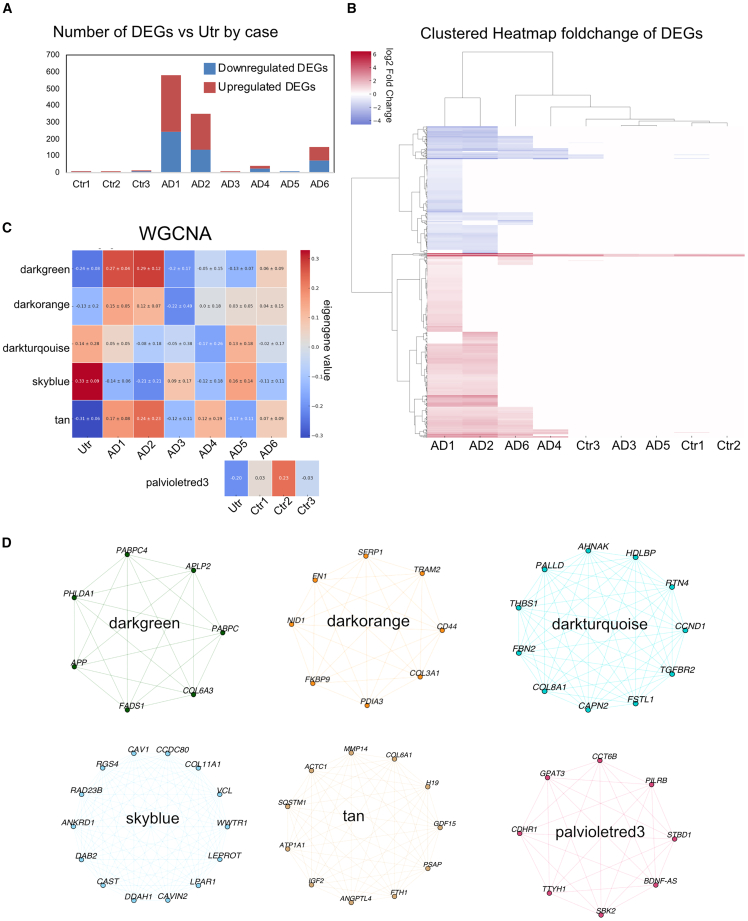
Heterogeneity in astrocytic gene expression response between AD cases iPSC-astrocytes were treated for 7 days with 35 ng/mL of tau in sarkosyl-insoluble fractions of AD cases (AD1–6) and equivalent volumes of control brains (Ctr1-3) and compared individually to untreated astrocytes after bulk RNA-sequencing. (A) Total number of up- (red) and down- (blue) regulated DEGs in astrocytes exposed to samples from individual AD and control cases, compared to untreated cells. (B) Hierarchical clustered heatmap showing all significant genes (*p* < 0.05) for astrocytes treated with each case compared to untreated. (C) Weighted correlation network analysis (WGCNA) heatmap depicting modules with most consistent expression changes across technical repeats, compared to untreated astrocytes. The strength of gene expression for each module is represented by its eigengene value representing trend of upregulation (red) or downregulation (blue) of gene in that module compared to untreated astrocytes. Each module displays a mean of 3 technical repeats ±SD. (D) Highest expressed genes for each module from (C) depicted in network diagrams.

We observed significant disparity in the number of DEGs across treatment conditions (Figures 6A and S6A). Control conditions (Ctr1-3) exhibited relatively low numbers of DEGs, with counts of 8, 5, and 15, respectively. In contrast, AD-treated conditions showed a wider range of DEG counts, from as few as 2 in AD5 to as many as 581 in AD1, indicating a substantial variation in gene expression responses to spiking of cells with AD tau aggregates, which may relate to specific post-translational modifications of tau. When assessing the similarity between treatment groups using the Jaccard index, the average similarity score among all AD treatment groups was approximately 0.130, while the control groups exhibited a higher average similarity score of approximately 0.333, indicating a more consistent gene expression response across control treatments. A hierarchical cluster analysis based on fold change of significant DEGs grouped AD1 and AD2 as unique from other treatment conditions (Figure 6B). Of the remaining AD treatments, AD6 and AD4 showing greatest similarity, while AD3 and AD5, the two AD cases that were unable to seed astrocytic tau in our long-term assay (Figure 3C), clustered more closely with controls (Ctr1-3).

Weighted gene co-expression analysis (WGCNA) identified 5 modules across AD tau-treated cells and one control module that were selected based on uniformity of eigengene values across technical replicates relative to untreated cells (Figure 6C). The highest expressed genes in each module are highlighted in network maps (Figure 6D) and expression heatmaps (Figure S6B).

The **darkorange** module contains genes involved in extracellular matrix interactions (*NID1*, *FN1*, and *COL3A1*)^49^^,^^50^ protein folding and ER stress response (*PDIA3*, *FKBP9*, and *TRAM2*),^51^^,^^52^^,^^53^ as well as *CD44*, which may have a role in mediating neuroinflammatory responses.^54^ AD1 and AD2 demonstrated the strongest deviation from untreated cells and had the highest eigengene values, whereas AD3 was similar to untreated cells, again indicating variability in the response to tau from different AD cases.

The **darkgreen** module was starkly upregulated in AD1 and AD2 compared to untreated cells, with only moderate differences between cells treated with tau from the other AD cases and untreated controls. This module contains genes associated with amyloid precursor protein processing (*APP* and *APLP2*)^55^ and lipid metabolism (*FADS1*).^56^ It also contains genes with functions in mRNA stability and translation (*PABC1* and *PABPC4*),^57^^,^^58^ and *PHLDA1* which is linked to microglia activation.^59^

The **tan** module contains genes involved in autophagy (*SQSTM1*),^60^^,^^61^ lysosome function and lipid metabolism (PSAP),^62^ extracellular matrix remodeling (*MMP14* and *COL6A1*),^63^^,^^64^ growth factors that influence cell growth, differentiation and survival (*IGF2)*^65^ whose expression has been noted to decrease in AD patients vs. controls, and which has been touted as a potential therapeutic target.^66^ Other hits included *GDF15*, a mitochondrial stress response molecule^67^ and *ANGPTL4*,^68^ iron metabolism (*FTH1*), which has been linked to ferroptosis in AD brain via single cell RNA-seq analysis,^69^ and ion transport across membranes (*ATP1A1*).^70^ Again, this upregulation was particularly pronounced when cells were treated with tau from AD1 and AD2, while cells treated with AD3 and AD5 extracts showed expression similar to untreated cells.

For the **darkturquoise** module, expression was reduced in the majority of AD treated astrocytes compared to untreated, except for AD5. This module again contains genes involved in extracellular matrix organization including THBS1,^71^ *FBN2*,^72^ and *COL8A1*, the latter being upregulated in an AD specific astrocyte cluster analysis.^73^ Other hits include genes involved in growth factor signaling such as *FSTL1* and *TGFBR2*.^74^^,^^75^ *RTN4* is within this module, a regulatory transmembrane protein implicated in neurodegenerative disease.^76^^,^^77^ *PALLD* encodes a cytoskeletal protein involved in cell shape and motility.^78^ Genes related to protein synthesis and trafficking (*HDLBP*^79^ and *AHNAK*^80^ were also detected. *CCND1* also present in this module, is important for cell cycle regulation and has been identified as a transcriptional regulator of reactive astrocytes,^81^ as well as *CAPN2*, a protease studied in AD models and with therapeutic potential.^53^^,^^82^

The **skyblue** module was also decreased when cells were treated with tau from most AD cases compared to untreated. It contains genes such as *CAV1*, *CAVIN2*, and *LPAR1*, important in cell signaling and membrane dynamics.^83^^,^^84^^,^^85^ The involvement of genes related to the extracellular matrix is again highlighted by *COL11A1*^86^ and *VCL*,^87^ reinforcing the importance of cell-matrix interactions. Additionally, *WWTR1*^88^ and *RGS4*^77^ underscore transcriptional regulation and signal transduction genes, with both previously noted as hub genes altered in AD progression. The module also showcases *DDAH1* and *CAST* for their contributions to protein metabolism,^89^^,^^90^ along with *DAB2*^91^ and *RAD23B*^92^ which have roles in endocytosis and nucleotide excision repair. There was a general decrease in expression across all AD cases compared to untreated cells, most profoundly for those treated with AD1, AD2, AD4, and AD6 tau, but not AD3 and AD5, suggesting that these genes are altered with pathological tau seeding and accumulation.

Finally, the **palevioletred3** module highlights genes upregulated following exposure to control brain extracts relative to untreated cells. The genes in this module are involved in various cellular processes including glycophagy (*STBD1)*,^93^ kinase activity (*SBK2)*,^94^ immune response regulation (*PILRB*)^95^ lipid biosynthesis *GPAT3*,^96^ and *BDNF-AS* which regulates the expression of *BDNF*.^97^

These insights underscore the complex and heterogeneous astrocyte responses to AD tau, spotlighting the significant role of distinct gene modules in AD progression and the potential influence of these genes on astrocyte physiology in response to pathological tau processing.

## Discussion

Astrocytic tau accumulations are characteristic of several primary tauopathies including progressive supranuclear palsy and corticobasal degeneration. Recent studies^14^^,^^98^ have also observed tau aggregates within astrocytes in AD brain, although their specific role in AD is not well understood. Consequently, several aspects require further exploration: the frequency and dynamics of astrocytic tau inclusions in AD brain, the capacity of astrocytes to eliminate these inclusions or contribute to tau spread, and the impact of such inclusions on astrocyte function. We make progress in illuminating some of these roles for astrocytes in AD using a relatively small number of AD cases. We show differences in astrocyte responses to tau from different AD cases, which likely reflects molecular heterogeneity of tau, giving some insights into the nuance required for interpreting astrocyte responses to tau in disease.

Using high-throughput immunofluorescence screening of temporal cortex, we show that astrocytes can be associated with pathological tau inclusions in the AD cases examined here, an observation which extended to one control case that showed early Braak staging, suggesting that astrocytes may develop tau inclusions early in disease. There was considerable variation in the amounts of astrocytic AT8 immunoreactivity observed between cases. This likely reflects variability in individual burden of tau pathology and astrocytic involvement, in keeping with other reports on disease heterogeneity.^4^^,^^7^ The extent of postmortem delay before tissue processing may also play a role in this variability, however, we did not detect a direct correlation between postmortem delay and AT8 abundance in our samples, and astrocytic AT8 immunoreactivity was significantly higher in AD relative to controls.

In AD brain, astrocytes come into contact with “ghost” tangles that exist in the extracellular spaces,^99^^,^^100^ and potentially internalize tau aggregates along with neuronal debris, as previously described.^17^ However, astrocytes also internalize isolated tau.^18^^,^^19^^,^^30^^,^^31^ To further explore responses of astrocytes to pathological tau, we optimized a human cell-based assay for seeding with AD brain derived sarkosyl-insoluble tau aggregates, considering that synthetic tau fibrils, with their distinct conformational structure,^20^ might elicit different cellular responses. These fractions were found to contain several other protein components, and unbiased cluster analysis distinguished between samples that induce tau seeding in astrocytes (AD1, AD2, AD4, and AD6) and those that do not (AD3 and AD5), suggesting that co-factors as well as post-translational modifications may influence tau seeding and potentially spread in diseased brain. Additional work is required to determine the relative contributions of tau and other components of the human brain extracts. In particular, future work should examine which of these proteins directly interacts with tau and may facilitate either tau seeding of endogenous astrocytic tau or tau clearance from astrocytes.

We found that astrocytes internalized tau aggregates at a relatively slow rate in comparison to studies involving microglia.^16^^,^^101^ Interestingly, the rate of tau uptake varied depending on the AD case, as did the propensity to seed endogenous tau aggregation. One possible explanation is that although AD tau fibrils share a common proteopathic core^5^ their post-translational modification profiles can significantly impact tau seeding.^7^^,^^25^ Notably AD3 and AD5, two cases we found to have slow rate of uptake and more efficient clearance by astrocytes, had unique phosphorylation profiles that distinguished them from other cases that we studied, including peptides phosphorylated at sites that have previously been shown to negatively correlate with tau seeding and disease progression.^7^ Moreover, the abundance of peptides modified at S262 was relatively low for AD5 compared to other cases, and phosphorylation at this site has shown positive correlation with tau seeding.^7^ Further, phosphosites S113 and S175, uniquely detected in AD3 and AD5 as well as S191 in AD5, were more common in a cluster of AD cases that had lower tau burden.^4^ While it has often been assumed that greater overall levels of phosphorylation promote tau aggregation, our work is in line with recent studies that show that tau aggregation is dependent on specific sequence modifications in tau,^7^^,^^102^ and that some modified sites can in fact inhibit aggregation.^24^ More work is required to carefully delineate how specific modification sites of tau can affect uptake, seeding, and downstream cell type response.

Emerging evidence supports the role of astrocytes in not only internalizing tau but also facilitating its spread to neighboring cells, thus propagating tau pathology.^17^^,^^31^ Our work further suggests that tau modifications impact tau uptake and seeding in astrocytes that may contribute to this process. Our work agrees with others that human astrocytes express *MAPT*^35^ and, therefore, provide a source of tau (albeit at low levels) for templated misfolding. Much as tau aggregation affects neuronal function, we found that tau accumulation in astrocytes affected astrocytic proteins such as GFAP and S100B, both of which are linked to AD progression.^103^^,^^104^^,^^105^ S100B localizes with tau in neuroblastoma cells, and can prevent tau seeding and limit liquid-liquid phase separation *in vitro.*^23^^,^^106^ However, S100B may also contribute to tau hyperphosphorylation through DKK-1 upregulation.^107^ Its persistence around internalized tau aggregates after exogenous tau removal in our assay indicates an insufficient capacity to prevent endogenous astrocytic tau seeding. GFAP, indicative of astrocyte reactivity,^41^ also showed a positive correlation with AT8 tau internalization, and may be sequestered by tau aggregates in the cytosol. Indeed, tau fibrils do not form as homogeneous aggregates in AD brain, and several studies have shown other proteins are recruited into larger fibrils with tau,^108^ as also suggested by our analysis of sarkosyl-insoluble fractions. S100B was not detected in our sarkosyl extracts, therefore, we believe these data reflect the sequestration of endogenous proteins by internalized tau aggregates. While GFAP was present in our sarkosyl extracts, we found higher GFAP levels around tau inclusions following the removal of sarkosyl extract from media and extended periods of culture (Figure S5), indicating a progressive accumulation of endogenous GFAP around remaining tau aggregates.

Taken together, molecular properties of tau, as defined through analysis of post-translational modifications, showed associations with both the rate of tau uptake and either seeding of endogenous tau or clearance by human astrocytes, as well as changes in astrocyte function indicated by gene expression changes. While postmortem delay (PMD) can have marked influences on tau phosphorylation, our findings suggest that PMD alone does not account for the observed variability in tau PTM profiles. These findings align with prior research indicating that heterogeneity in AD tau modifications is driven by intrinsic disease-related factors, including tau strain variation and individual differences in tau post-translational modifications.^4^^,^^7^ While PMD remains a potential confounder, our data suggest that tau modifications are largely dictated by disease processes rather than postmortem degradation alone.

Astrocyte reactivity has wide-ranging implications for AD progression, with unique signatures in AD brain.^109^ Responses to Aβ and tau pathology can differ,^110^ and efforts are ongoing to resolve transcriptional changes during the spatial and temporal progression of AD pathology.^111^^,^^112^ Our RNA-seq analysis revealed highly significant DEGs unique to the AD group, including those related to the complement system (*CX3CL1*) and mitochondrial function and autophagic processes (*HGMCS2*), both previously implicated in AD pathology.^42^^,^^43^^,^^47^^,^^48^ While control brain extracts induced some overlapping gene expression which might reflect effects of other protein components in the sarkosyl-insoluble fraction, AD brain extracts produced a higher number of DEGs with a larger effect size. Again, variability in astrocyte gene changes was noted depending on the case used. Tau from AD3 and AD5, distinguished by their PTM profiles, showed slower uptake rates and did not seed further tau aggregation, and exhibited fewer DEGs. WGCNA analysis unveiled gene modules intersecting with cardinal features of AD pathology. The darkgreen module accentuated genes involved in amyloid precursor protein processing, including *APP* and *APLP2*, potentially linking tau uptake-induced astrocytic gene expression alterations to amyloid-beta pathology. This module also contained genes related to lipid metabolism, mRNA stability, and microglia activation.

We also observed changes to genes related to protein folding and endoplasm reticulum (ER) stress response after tau uptake, another mechanism implicated in AD onset and development.^53^ *PDIA3*, an ER isomerase expression is altered in 3xTg-AD mice,^51^ and *FKBP9* part of a class of chaperones linked to AD progression,^52^ has been directly implicated in a prion seeding assay *in vitro.*^113^ In addition, *TRAM2* is a member of the translocon that is involved in the posttranslational processing of proteins at the ER membrane.^114^

Several modules demonstrated genes related to autophagy, lysosome and proteasome function, emphasizing the protein clearance pathways altered as astrocytes process tau aggregates. These included *HMGCS2*, *SQSTM1*,^60^ and *PSAP*, the latter being important for dopaminergic lipid homeostasis in a Parkinson’s model.^62^ Other modules linked genes related to protein metabolism such as *CAST* (calpastatin), *CAPN2* (calpain-2), *HDLBP*,^79^ and *AHNAK*.^80^ Calpain activity has been shown to be upregulated prior to tau phosphorylation and loss of synaptic cells in AD brain,^115^ and calpain-2 specifically can create tauopathy-associated tau fragments.^116^ Inhibition of calpastatin degradation reduced neuropathology in mouse models of Huntington’s,^90^ highlighting this pathway as a potential therapeutic target to reduce neuronal death.

Multiple modules contained genes integral to ECM organization and maintenance, including *MMP14*, *COL6A1*, *NID1*, *FN1*, *THBS1*, *FBN2*, *COL8A1*, *COL3A1*, *COL11A1*, and *VCL*.^49^^,^^50^^,^^63^^,^^64^^,^^71^^,^^72^^,^^86^^,^^87^ This suggests astrocyte involvement in restructuring the brain’s extracellular environment in response to AD tau pathology. Components of the ECM are known to form part of amyloid structures in AD brain,^108^ and ECM remodeling can influence the distribution and aggregation of Aβ.^117^ The rapid alterations in ECM related genes in our assay suggest that astrocytes play a role in this dysfunction.

Overall, our study reveals that astrocytes efficiently internalize AD associated tau aggregates but process them in different ways depending on characteristics of the tau itself and/or co-factors. We also show that the expression of endogenous astrocytic tau may facilitate tau seeding in astrocytes during AD progression. We have identified that such uptake significantly influences astrocyte gene expression, pinpointing several genes previously linked with AD progression. This implies that the internalization of tau may prompt disease-associated astrocyte phenotypes, which likely disrupt interactions of astrocytes with neurons and other glial cells. Together, this study underscores the significance of understanding the diverse effects of tau on astrocytes within patient cohorts, distinctions crucial for developing successful therapies targeting astrocytic functions.

### Limitations of the study

This study has several limitations. The postmortem tissue was obtained from both male and female donors with an approximate ratio of 2 male:1 female case for the AD and control groups. There was no obvious effect of sex on experimental findings, but this cannot be definitely proven with the limited sample size used here. Similarly, the iPSC line used to generate astrocytes was from a male donor. For both the human postmortem brain tissue and iPSC line gender, ancestry, race, and ethnicity were unknown. Future studies are required to understand any influence of these characteristics on the results reported here. In addition, astrocytes were cultured in isolation and it will be important for future studies to determine the dynamics of tau processing in multiple cell types using co-cultures, organoids, or animal models. Similarly, future work should attempt to determine the nature of tau seeding observed, including if endogenous tau adopts properties in common with the exogenously AD brain aggregates, demonstrating templated misfolding. Additional work is also required to determine the mechanism by which tau is internalized by astrocytes and the nature of any processed tau that is subsequently released. It will also be essential in future studies to determine if it is indeed molecular properties of tau, or co-factors in the sarkosyl insoluble fraction that are driving differences in the handling of tau by astrocytes and effects on the astrocyte transcriptome.

## Resource availability

### Lead contact

Further information and requests for resources and reagents should be directed to and will be fulfilled by the lead contact, Wendy Noble (w.noble2@exeter.ac.uk).

### Materials availability

This study did not generate new unique reagents. Further information and requests for resources and reagents should be directed to and will be fulfilled by the lead contact, Wendy Noble (w.noble2@exeter.ac.uk).

### Data and code availability

• Data: All data generated or analyzed during this study are included in this published article and its supplementary files 1 and 2. The mass spectrometry proteomics data have been deposited to the ProteomeXchange Consortium via the PRIDE^118^ partner repository with the dataset identifier PXD056614. Raw FASTQ files have been deposited at the National Center for Biotechnology Information (nih.gov) Sequence Read Archive (SRA) database with Bioproject ID:PRJNA1171874. Summary data for main and supplementary figures is available on FigShare (https://doi.org/10.6084/m9.figshare.28955612).
• Code: We generated custom code for hierarchical cluster analysis. The code can be found in supplemental information. Further information and requests should be directed to the lead contact, Wendy Noble (w.noble2@exeter.ac.uk).
• All other items: Any requests for additional information should be directed to the lead contact, Wendy Noble (w.noble2@exeter.ac.uk).

## Acknowledgments

We thank the late Professor Peter Davies for tau antibodies, Dr George Chennell of the Wohl Cellular Imaging Center at King’s College London for technical support, and the London Neurodegenerative Disease Brain Bank. This work was funded by 10.13039/501100002283Alzheimer’s Research UK (ARUK-PG2019A-004) to WN and B.G.P.-N., Van Geest Charitable Foundation funding to B.G.P.-N., an 10.13039/501100000265MRC Doctoral Training Partnership studentship to W.N., B.G.P.-N., and M.J.R., and a London 10.13039/501100002283Alzheimer’s Research UK Network Center pump-prime award to M.J.R. and M.L.S. The London Neurodegenerative Disease Brain Bank receives funding from the 10.13039/501100000265Medical Research Council and the Brains for Dementia Research program, jointly funded by 10.13039/501100002283Alzheimer’s Research UK and 10.13039/501100017506Alzheimer’s Society. For the purpose of open access, the author has applied a Creative Commons Attribution (CC BY) licence to any Author Accepted Manuscript version arising from this submission.

## Author contributions

W.N., B.G.P.-N., M.J.R., and M.L.S. designed the study and W.N. and B.G.P.-N. supervised the research. M.J.R. planned and performed most experiments and analyzed data with help from M.L.S., S.L., and C.T. with critical input from D.P.S. All authors read, edited, and approved the final manuscript.

## Declaration of interests

The author declare that they have no competing interests.

## STAR★Methods

### Key resources table

**Table:** 

REAGENT or RESOURCE	SOURCE	IDENTIFIER
**Antibodies**		

Mouse anti-phosphorylated tau (AT8)	Thermo Fisher Scientific	Cat# MN1020; RRID: AB_223647
Rabbit anti-tau	Agilent/Dako	Cat# A0024, RRID: AB_10013724
Rabbit anti-S100 Beta	Proteintech	Cat# 15146-1-AP; RRID:AB_2254244
Chicken anti-GFAP	Invitrogen	Cat# PA1-10004; RRID: AB_1074620
Rabbit anti-GFAP	Agilent/Dako	Cat# Z0334, RRID:AB_10013382
Mouse anti-GFAP	Santa Cruz Biotechnology	Cat# sc-33673; RRID: AB_627673
Mouse monoclonal anti-pTau (PHF1)	Peter Davies, Albert Einstein College of Medicine	Cat# PHF1; RRID: AB_2315150
Rabbit anti-tau (4R)	Cell Signaling Technology	Cat# 30328; RRID: AB_2267689
Mouse anti-beta actin	Abcam	Cat# 8226; RRID: AB_306371
Mouse anti-GAPDH	Santa Cruz Biotechnologies	Cat# sc-32233; RRID: AB_627679
Goat anti-Mouse, Alexa Fluor 680	Thermo Fisher Scientific	Cat# A-21057; RRID: AB_2535723
Goat anti-Rabbit, Alexa Fluor 568	Thermo Fisher Scientific	Cat# A-11011; RRID: AB_143157
Goat anti-Rabbit, Alexa Fluor 488	Thermo Fisher Scientific	Cat# A-11034; RRID: AB_2576217
Goat anti-Chicken, Alexa Fluor 568	Thermo Fisher Scientific	Cat# A-11041; RRID: AB_2534098
IRDye® 680RD Donkey anti-Rabbit	LI-COR Biosciences	Cat# 926-32214; RRID: AB_621846
IRDye® 680RD Goat anti-Mouse	LI-COR Biosciences	Cat# 926-68070; RRID: AB_10956588
Alpaca anti ms (IgG) 488	Proteintech	Cat# sms1AF488-1; RRID: AB_2827578
Alpaca anti ms (IgG2b) 568	Proteintech	Cat# sms2bAF568-1; RRID: AB_2827582
Alpaca anti rb IgG 647	Proteintech	Cat# srbAF647-1; RRID: AB_2827587

**Chemicals, peptides and recombinant proteins**		

Poly-D-Lysine	Thermo Fisher Scientific	Cat# A3890401
RevitaCell Supplement	Thermo Fisher Scientific	Cat# A2644501
Astrocyte Medium	ScienCell	Cat# 1801
DMEM/F12 Medium	Thermo Fisher Scientific	Cat# 11320033
Neural Induction Medium	Thermo Fisher Scientific	Cat# A1647801
Geltrex	Thermo Fisher Scientific	Cat# A1413201
StemFlex Medium	Thermo Fisher Scientific	Cat# A3349401
Versene Solution	Thermo Fisher Scientific	Cat# 15040066
StemPro Accutase Solution	Thermo Fisher Scientific	Cat# A1110501
1-β-D-Arabinofuranosylcytosine (AraC)	Sigma-Aldrich	Cat# 251010
Trizol Reagent	Invitrogen	Cat# 15596026
Complete mini Protease inhibitor EDTA free	Roche	Cat# 11836170001
PhosStop phosphatase inhibitor	Roche	Cat# 4906845001
N-Lauroylsarcosine sodium salt solution (Sarkosyl)	Sigma-Aldrich	Cat# L7414

**Critical commercial assays**		

Maxima H Minus First Strand cDNA Synthesis Kit, with dsDNase	Thermo Fisher Scientific	Cat# K1681
Phasemaker Tubes	Invitrogen	Cat# A33248
Human SERPINA3(Alpha-1-antichymotrypsin) ELISA Kit	FineTest	Cat# EH0570

**Experimental model: Cell line**		

iPSC line derived from neurotypical male	Srivastava Lab, King’s College London, UK^119^	N/A

**Biological samples**		

Human postmortem brain tissue (temporal cortex) (See Table S1)	London Neurodegenerative Diseases Brain Bank/Brain for Dementia Research at King’s College London	N/A

**Software and algorithms**		

Harmony v4.9	Perkin Elmer	https://content.perkinelmer.com/lab-products-and-services/resources/software-downloads.html
GraphPad Prism v9	GraphPad Software Inc	https://www.graphpad.com/resources
ImageStudio Lite v5.2.5	LI-COR Biosciences Ltd	https://www.licor.com/bio/image-studio/resources
Visiopharm v2023.01	Visiopharm UK Limited	https://visiopharm.com/research-pathology-image-analysis-software/
Custom code for hierarchical cluster analysis	Python.org	Supplemental file (Methods S1).

**Deposited data**		

Mass spectrometry data	ProteomeXchange Consortium	PXD056614https://proteomecentral.proteomexchange.org/?search=PXD056614
RNA sequencing data	NIH sequence read archive (SRA database)	PRJNA1171874 https://www.ncbi.nlm.nih.gov/sra/?term=PRJNA1171874
Summary data for main and supplementary figures	Figshare	https://doi.org/10.6084/m9.figshare.28955612

### Experimental model and study participant details

#### Ethics approval and consent to participate

All donors had provided written informed consent for the use of postmortem brain for research purposes to the London Neurodegenerative Disease Brain Bank. All human tissue collection and processing were carried out under the regulations and licensing of the Human Tissue Authority, and in accordance with the UK Human Tissue Act, 2004. The iPSC line used in this study (CTR_M3_36S) was received as a donation from Prof. Deepak Srivastava at King’s College London. Consent for storage of tissue and iPSCs and explicit consent for use in subsequent research was obtained by the original lab, following the guidelines of the UK Human Tissue Act 2004. Specific Research Ethics Committee approval is not a legal requirement for generating iPSCs from human donor tissue.

#### Postmortem human brain

Postmortem human brain samples from neuropathologically confirmed AD cases and age-matched controls (with no neurological condition) were requested from the London Neurodegenerative Diseases Brain Bank/Brains for Dementia Research at King’s College London, research ethics committee (REC) reference WA/0124. Tissue was obtained from both male and female donors, ancestry, race and ethnicity being unknown. The ratio of male: female is 2:1 for both AD (*n* = 6) and control (*n* = 3) groups. Key characteristics are shown in Table S1. Samples were taken from the temporal cortex by an experienced pathologist. Pathological diagnosis was obtained by routine pathological analysis after donation to the brain bank and conducted by an experienced pathologist.

#### Human iPSC line

The iPSC line used in this study (CTR_M3_36S) was received as a donation from Prof. Deepak Srivastava at King’s College London, previously generated from hair-root derived keratinocytes of a healthy control male, as described in Cocks et al.^120^ Ancestry, race and ethnicity are unknown. The line was created with a CytoTune-iPS 2.0 Sendai Reprogramming Kit (Cat# A16517; Invitrogen). This line was previously characterized for stable karyotype, differentiation into 3 germ layers, and efficient clearance of reprogramming transcription factors.^119^ iPSC were routinely screened, and were negative, for mycoplasma contamination.

### Method details

#### Isolation of tau from postmortem human brain

Tau was extracted from postmortem human brain tissue using a protocol modified from Greenberg & Davies.^121^ Briefly, brain tissue samples were homogenized at 100 mg/mL in Tris-Buffered Saline (TBS: 50 mM Tris-HCl, 150 mM NaCl, pH 7.4) containing 2 mM EGTA, 10% (w/v) sucrose, Complete Mini Protease Inhibitor Cocktail, and PhosSTOP phosphatase inhibitors (Roche, Basel, Switzerland). Homogenization was performed using a Tissue Master homogenizer (Omni International, USA). Sarkosyl (Sigma-Aldrich, St. Louis, MO, USA) was added to the homogenate to a final concentration of 1% (v/v), and samples were agitated at room temperature for 30 min. Samples were centrifuged at 136,000 x g for 1 h at ambient temperature using a Beckman Coulter Optima MAX-XP Ultracentrifuge with a TLA-55 rotor (Beckman Coulter, CA, USA). The resulting sarkosyl-insoluble pellet was washed in homogenization buffer containing 1% sarkosyl, re-centrifuged, and then resuspended in sterile Dulbecco’s PBS (Thermo Fisher Scientific, MA, USA). The final sarkosyl-insoluble tau suspension was sonicated using a Bandelin Sonopuls HD 2070 (BANDELIN electronic GmbH & Co, Berlin, Germany). Sonication settings were 3 × pulses at 10% power on ice. For quantification, tau was analyzed using SDS-PAGE and western blotting. Known concentrations of recombinant human tau (Human Tau Protein Ladder 6 isoforms; Sigma-Aldrich, MO, USA; Cat# T7951) were loaded alongside extracted sarkosyl insoluble samples (National Diagnostics, Hull, UK). After SDS-PAGE and immunoblotting, the abundance (intensity) of tau present in the entire lane of each sample was quantified. Tau content in postmortem brain samples was compared to a standard curve generated from the signals obtained from recombinant human tau of known concentration, using the formula: =y−c/m , where x is the amount of tau, y is the band intensity, c is the y-intercept, and m is the slope of the standard curve. Based on these results, tau protein levels across postmortem cases was normalized prior to mass spectrometry.

#### iPSC differentiation into astrocytes

Astrocytes were differentiated from control iPSCs using a well-characterized protocol.^36^ iPSCs were cultured and differentiated into neural progenitor cells (NPCs) using the Gibco PSC Neural Induction (NI) Medium system (Thermo Fisher Scientific). When iPSCs reached 15–25% confluency, the medium was replaced with Neural Induction Medium (NI medium), consisting of Neurobasal Medium and Neural Induction Supplement (1:50). Cells were maintained in NI medium at 37°C in a non-hypoxic incubator (20% O_2_, 5% CO_2_) for 7 days. Medium changes were performed as follows: on day 3, fresh NI medium was added at 2.5 mL per well; on day 5, the volume was doubled to 5 mL per well; on day 7, the medium was refreshed. By day 8, cells reached confluency and were ready for passaging. For passaging, medium was aspirated, and cells were dissociated using room temperature StemPro Accutase solution (Thermo Fisher Scientific, Cat# A1110501), transferred to a tube containing prewarmed DMEM/F-12 medium (Thermo Fisher Scientific, Cat# 11320033) and centrifuged at 190 x g for 2 min. The cell pellet was resuspended in Neural Expansion Medium (NE medium), a 1:1 mix of Neurobasal Medium and Advanced DMEM⁄F-12 medium with Neural Induction Supplement. RevitaCell supplement (1:100, Thermo Fisher Scientific, Cat# A2644501) was added to NE medium to enhance cell survival during passaging. Cells were replated at a 1:3 ratio on Geltrex-coated 6-well plates (Thermo Fisher Scientific, Cat# A1413201) and maintained in NE medium. The medium was refreshed every 48 h. NPCs were passaged four times before being used for further differentiation or cryopreserved for future use.

NPCs were differentiated into astrocytes using Astrocyte Medium (ScienCell, Cat# 1801). NPCs were plated at a low density (15,000 cells/cm^2^) on Geltrex-coated 6-well plates (Thermo Fisher Scientific, Cat# A1413201) in Neural Expansion Medium (NE medium) with added RevitaCell supplement (1:100, Thermo Fisher Scientific, Cat# A2644501). After 24 h, the medium was replaced with Astrocyte Medium, which contains basal medium, fetal bovine serum (1:50), and astrocyte growth supplement (1:100). The medium was refreshed after another 24 h and subsequently every 48 h at 2.5 mL per well. By approximately day 6, when cells reached 90% confluency, they were passaged at the same density. Starting from day 30, fetal bovine serum was removed from the Astrocyte Medium. Cells were passaged at a 1:3 ratio approximately once a week. Astrocytes were maintained in these conditions up to day 60, at which point they were either cryopreserved or used for experimental assays.

#### Liquid chromatography-tandem mass spectrometry (LC-MS/MS)

Samples were submitted to the CEMS Proteomics Facility (James Black Center, King’s College London) for analysis by high resolution Orbitrap tandem mass spectrometry coupled to liquid chromatography for protein identification. Sarkosyl-insoluble pellets from the temporal cortex of AD and control patients were sonicated in DPBS, and protein concentration was determined using a BCA assay. Samples (30 μL) were prepared for analysis by high-resolution Orbitrap tandem mass spectrometry. For enzymatic digestion, 70 μL of 50 mM triethylammonium bicarbonate (Cat. No. T7408; Merck) was added to each sample to a total volume of 100 μL. After brief vortexing, 11 μL of 50 mM dithiothreitol (DTT, Cat. No. D5545; Merck) was added, followed by incubation at 56°C for 30 min. For alkylation, 12 μL of 200 mM iodoacetamide (Cat. No. I1149; Merck) was added and incubated at room temperature in the dark for 20 min. The reaction was quenched with 5 μL of 50 mM DTT. Finally, 1 μg of trypsin (Cat. No. 000000011047841001; Merck) was added, and samples were incubated overnight at 37°C. Peptides were dried using a Speedvac (Thermo Fisher Scientific), resuspended in 0.1% trifluoroacetic acid (TFA), and purified using C18 spin columns (#89852; Thermo Fisher Scientific). Peptides were eluted in 50% acetonitrile/0.1% TFA, dried again, and stored at −80°C. Peptides were resuspended in MS sample buffer (2% acetonitrile:0.05% trifluoroacetic acid) and injected for analysis on a U3000 UHPLC NanoLC system (Thermo Fisher Scientific, UK). Peptide separation was performed on a 75 mm C18 Pepmap column (50 cm length) using a linear gradient of 80% acetonitrile in 0.1% formic acid, with a flow rate of 250 nL/min over 60 min. Electrospray ionization was performed using an Orbitrap Fusion Lumos (Thermo Fisher Scientific, UK). Full MS scans (FTMS1) were acquired at a resolution of 120,000 over an m/z range of 375–1800. MS/MS fragmentation (ITMS2) used collision-induced dissociation with a 3-s cycle time, dynamic exclusion of 35 s, and isolation width of 1.6 m/z. The AGC target was set to 4.0e5 for FTMS1 and 1.0e4 for ITMS2, with a maximum injection time of 35 ms.

#### Immunohistochemistry of human brain tissue

Formalin fixed, paraffin-embedded brain sections (7 μm) from postmortem human samples were deparaffinized, rehydrated, and antigen retrieval conducted using a sodium citrate buffer (10 mM trisodium citrate dihydrate, 0.05% Tween 20, pH 6.0). After washing in Tris-Buffered Saline (TBS), sections were blocked with 10% normal goat serum (NGS) in TBS for 1 h at room temperature. Primary antibodies used were anti-S100B (1:500, Proteintech, Cat# 15146-1-AP), anti-GFAP (1:500, Invitrogen, Cat# PA1-10004), and anti-phosphorylated tau (AT8; 1:300, Thermo Fisher Scientific, Cat# MN1020). Slides were incubated with primary antibodies overnight at 4°C. After washing in TBS, secondary antibodies (Goat anti-Rabbit Alexa Fluor 488, Thermo Fisher Scientific, Cat# A-11034; Goat anti-Chicken Alexa Fluor 568, Thermo Fisher Scientific, Cat# A-11041; Goat anti-Mouse Alexa Fluor 680, Thermo Fisher Scientific, Cat# A-21057) were applied for 1 h at room temperature in the dark.To reduce autofluorescence, slides were treated with Sudan Black solution, followed by washing in TBS. Nuclei were counterstained with Hoechst 33342 (Thermo Fisher Scientific, Cat# H3570) before coverslipping with ProLong Gold Antifade Mountant (Thermo Fisher Scientific, MA, USA).

#### RNA extraction, RT and qPCR

Total RNA was extracted from astrocytes using TRIzol Reagent (Cat# 15596026) and Phasemaker Tubes (Cat# A33248), following the manufacturer’s protocol with the addition of glycogen to enhance RNA pellet recovery. Purified RNA (1–5 μg) was reverse transcribed into complementary DNA (cDNA) using the Maxima H Minus First Strand cDNA Synthesis Kit with dsDNase (Cat# K1681), following the manufacturer’s instructions without random hexamer primers. RT-qPCR was performed using PowerUp SYBR Green Master Mix on a QuantStudio 7 Flex Real-Time PCR System. For 96-well PCR plates, each reaction contained 10 μL of master mix, 2 μL of 5 μM primers (combined forward and reverse), 2 μL of cDNA, and 8 μL of nuclease-free water, totaling 20 μL per reaction. For 384-well plates, reaction volumes were halved to 10 μL.

#### iPSC-astrocyte exposure to human tau aggregates

Day 60 astrocytes were plated as single cell suspensions at low density (6000 cells/cm^2^) in Astrocyte medium (ScienCell, CA, USA) with RevitaCell supplement (1:100) and 5 mM cytosine arabinoside (AraC) to reduce proliferation. After 24 h, media was changed for Astrocyte medium with 5 mM AraC only. After a further 48 h, media was changed to Astrocyte medium only. Sarkosyl-insoluble AD tau or equivalent control brain fractions were added to Astrocyte medium at a tau concentration of 35 ng/mL. For control samples with low tau concentration, a median equivalent of AD sample volumes was used. Media was exchanged for the same after 3 days. Astrocytes were incubated in spiked medium for 1, 3, 5 or 7 days for 7-day, and all astrocytes were fixed at the same timepoint in ice-cold methanol for 5 min at −20°C. For RNA and ELISA analysis at day 7, media was collected and cells lysed in TRIzol reagent as described below. For astrocyte characterization, cells were lysed or fixed at day 70. To study tau handling beyond 7 days, wells were aspirated, washed and fresh Astrocyte medium added every 7 days. Plates were fixed after an additional 14 or 28 days (Figure 3A).

#### Imaging of tau handling by iPSC-astrocytes

iPSC-derived astrocytes were cultured on PhenoPlate 96-well microplates (PerkinElmer, MA, USA; Cat# 6055302) were fixed by replacing the medium with ice-cold methanol and placing at −20°C for 5 min. Non-specific antibody binding was blocked with 5% bovine serum albumin (BSA; Cat# A9418 Sigma-Aldrich, MO, USA) in DPBS containing calcium and magnesium, for 1 h at ambient temperature. Primary antibodies against phosphorylated tau (AT8; 1:500, Thermo Fisher Scientific, Cat# MN1020), GFAP (1:500, Santa Cruz Biotechnology, Cat# sc-33673), and S100B (1:500, Proteintech, Cat# 15146-1-AP) were incubated along with nano secondary antibodies of alpaca anti-mouse IgG1 Alexa Fluor 488 (1:1000, Proteintech, Cat# sms1AF488-1), anti-mouse IgG2b Alexa Fluor 568 (1:1000, Proteintech, Cat# sms2bAF568-1), and anti-rabbit IgG Alexa Fluor 647 (1:1000, Proteintech, Cat# srbAF647-1) overnight at 4°C. Cells were washed and nuclei were counterstained with Hoechst 33342 (Thermo Fisher Scientific, Cat# H3570). Imaging was performed using a confocal Opera Phenix high-content screening system (PerkinElmer, MA, USA). A 20X water objective lens (NA 1.0) was used, with laser excitation at 385 nm, 488 nm, 561 nm, and 640 nm for detecting Hoechst, Alexa Fluor 488, 568, and 647 fluorophores. Images covered up to 25 fields per well, with 15 z-stacks at 0.8 μm intervals.

#### SDS-PAGE and immunoblotting

Protein samples were prepared in 2X Sample Buffer or 4X NuPage Sample Buffer with reducing agent, then denatured by heating at 95°C for 5 min (or at 70°C for 10 min for NuPage samples). Proteins were separated on NuPAGE Bis-Tris 4-12% precast gels in the XCell SureLock Mini-Cell system using MOPS-SDS or MES-SDS running buffer, at a constant voltage of 120V. A protein ladder, Precision Plus Protein WesternC Blotting Standards (Bio-Rad, CA, USA), was loaded alongside the samples for molecular weight reference. Following electrophoresis, proteins were transferred onto Amersham Protran 0.45 μm nitrocellulose membranes (Cytiva, Amersham, UK) using the XCell II Blot Module in transfer buffer (2 mM Tris-Base, 192 mM glycine, 20% methanol) at a constant 0.3 A for 1 h. Non-specific binding was blocked with LI-COR TBS Blocking Buffer (LI-COR Biosciences, Lincoln, NE, USA) for 1 h at room temperature. Primary antibodies used for detection were PHF1 (pSer396/404) tau (1:1000, Peter Davies, Cat# PHF1; RRID: AB_2315150) and anti-tau (Dako, 1:1000, Agilent, Cat# A0024; RRID: AB_10013724). Membranes were incubated with primary antibodies overnight at 4°C, followed by washing in TBS-T (Tris-buffered saline with 0.1% Tween 20). Secondary antibodies were IRDye 680RD (1:10,000, LI-COR Biosciences, Cat# 926–68071) and IRDye 800CW (1:10,000, LI-COR Biosciences, Cat# 926–32211) for detection. Membranes were incubated with secondary antibodies for 1 h at room temperature, followed by washing in TBS-T.

#### RNA sequencing

Cells were washed to remove cellular debris and lysed with TRIzol Reagent (cat# 15596026) at approximately 0.4 mL reagent per 1 x 10^5^ cells. Total RNA was extracted from lysates using Phasemaker Tubes (Cat# A33248), following the manufacturer’s protocol with the addition of glycogen to enhance RNA pellet recovery. The RNA concentration and purity of the resulting samples was determined using the NanoDrop One/OneC Microvolume UV-Vis Spectrophotometer to ensure absorbance ratio of A280/A260 of approximately 2.0 and A260/A230 ratio above 2.0. Nanodrop spectrophotometry confirmed A260/230 ratios indicative of pure RNA. Extracted RNA was sent on dry ice to Genewiz by Azenta Life Sciences in Takely, UK for further RNA QC, library preparation (Illumina RNA with PolyA selection), sequencing (Illumina NovaSeq 2x150bp, 350M read pairs). Azenta provided a basic analysis package which included QC report, FASTQ files, data QC, trimming, mapping, differential gene expression, alternative splicing and gene ontology analysis. RNA integrity was further validated by Genewiz (Azenta) using Bioanalyzer/TapeStation QC before sequencing. No RNA degradation was observed in any samples.

### Quantification and statistical analysis

#### Image analysis

Slides of human brain tissue were scanned using an Olympus VS200 Research Slide Scanner equipped with a high-resolution digital camera and fluorescence capabilities, using a 40X objective lens. The system was set to detect Hoechst, Alexa Fluor 488, Alexa Fluor 568, and Alexa Fluor 680 channels with optimal exposure times to avoid bleed-through and cross-talk between fluorophores. Using Visiopharm software, regions of interest were manually selected in the gray matter. A nuclei detection protocol was used to identify cells, followed by the segmentation of cell boundaries to encompass the entire cell body. Positive GFAP and S100B fluorescence were used to differentiate astrocytes from other cell types. AT8 fluorescence intensity was specifically quantified within astrocytes to assess tau content, along with GFAP and S100B intensities. Data were exported in Excel format for further statistical analysis using GraphPad Prism.

Quantification of tau uptake by iPS-astrocytes was conducted using Harmony software (PerkinElmer, MA, USA). An analysis pipeline was created to quantify the proportion of dead or dying cells in each well. This was based on the appearance of nuclear staining in these wells. A linear classification system that utilises the pre-calculated size, intensity and roundness of Hoechst-stained nuclei was trained by machine learning to split cells into “healthy” and “dead or dying cells”.^40^ Astrocyte cell bodies were segmented using the ‘Find Cytoplasm’ function, identifying S100B and GFAP-positive cells. AT8 fluorescence intensity (maximum and average) was measured within GFAP-positive astrocytes to assess tau uptake. The ‘Find Region’ function was optimized to detect larger tau aggregates, enabling the calculation of volume and intensity properties for AT8, GFAP, and S100B channels. These parameters allowed for detailed analysis of tau uptake and astrocyte characteristics.

#### Mass spectrometry

Raw mass spectrometry data were processed into peak list files using Proteome Discoverer (ThermoScientific; v2.5). The raw data file was searched using the Sequest^122^ search algorithm against the Uniprot Human Taxonomy database (51,829 entries) and a bespoke database containing 6 tau isoforms of the human CNS (P10636-2, P10636-6, P10636-4, P10636-7, P10636-5, P10636-8). Database searching was performed at a stringency of 1% FDR including a decoy search. Posttranslational modifications for carbamidomethylation (C, static), oxidation (M, variable) and phosphorylation (S, T & Y; variable) were included in the database search. The database output file was uploaded into Scaffold software (version 5.1.2; www.proteomesoftware.com) for visualization and manual verification. The spectra of tau phosphorylation sites discovered through database searching were manually verified. To determine the relative abundance of tau peptides that were modified by phosphorylation, the ratio of modified to unmodified peptides were calculated for each phosphorylation site using their precursor ion abundance. If more than one peptide with the same phosphorylation site was detected, these precursor ion abundance values were combined and compared to unmodified peptides of the exact match. For modified peptides with no equivalent unmodified peptide and to ensure a value for each count, a small pseudo-constant at 1/10^th^ of the smallest non-zero abundance for unmodified peptides were used and applied to determine an adjusted ratio for each phosphorylation site (adjusted ratio = (modified precursor abundance)/(unmodified precursor abundance + pseudo-count). The adjusted ratio was normalized between 0 and 1 for each case and these values were plotted as a heatmap to represent the relative abundance of each phosphorylation site in each sample. For cluster analysis, normalized values of phosphorylation sites were converted to a binary scale, where any non-zero value was designated as '1' (presence) and zeros were maintained as '0' (absence). Hierarchical clustering was performed on the binary-transformed data to explore the patterns of phosphorylation site presence across different samples. The analysis was conducted using Python’s Seaborn and Matplotlib libraries. The Ward’s method was employed for clustering, which minimizes the variance within each cluster. The Euclidean distance metric was used to quantify the dissimilarity between the data points. Database searching of via the Uniprot Human Taxonomy database (51,829 entries) was used to determine the most abundant proteins in AD and control brain-derived sarkosyl-insoluble fractions by the precursor ion abundance. The mean of the Log2 of the precursor ion abundance was ranked for each protein and the top 20 for each sample were combined for a total of 43 proteins. Using Python, the protein abundance data were standardized using the StandardScaler function from the scikit-learn library to normalize the values. Hierarchical clustering was performed using the Ward’s method with the linkage function from the scipy library, minimizing within-cluster variance. The clustering results were visualized through a dendrogram and a heatmap using a custom python script (see Supplementary data file). The dendrogram was created using scipy, while the heatmap was generated using the seaborn.clustermap function, both incorporating Ward’s method and Euclidean distance. Data visualization was further enhanced using matplotlib. To compare AD (*n* = 6) and control groups (*n* = 3), the mean of the log2 precursor ion abundance for all detected proteins and fold change between the two groups was calculated and significant differences determined by an unpaired t test. The top 20 most significantly altered proteins were plotted in a bar chart.

#### Western blotting

Blots were visualized using the Odyssey CLx Imaging System (LI-COR Biosciences), and band intensities were quantified using Image Studio Lite software (LI-COR Biosciences). Intensities of proteins of interest were normalised to abundance of a housekeeping protein in the same sample, details of which are given in figure legends.

#### rtPCR

Gene expression analysis following rtPCR was conducted using the comparative CT method, normalized to two internal control genes, *β-ACTIN* and *GAPDH*, relative to a control sample. The fluorescence threshold was set automatically using QuantStudio Real-Time PCR Software v1.7.1. No template controls (NTC) were included to detect contamination or primer-dimer formation, and ‘Undetermined’ Ct values in NTCs were confirmed before analyzing sample data.

#### RNA sequencing

Three independent tau uptake experiments were performed to generate samples for RNAseq analysis, and data was averaged for analysis. Data was subsequently analyzed for comparison of differentially expressed genes (filtered by adjusted *p* value of <0.05) either by pooled AD and Control treated groups against untreated, or as individual cases against untreated. A custom Python script (see Supplementary data file) was used to generate the differential gene expression heatmap. Log2 fold change values for significantly altered genes were merged across treatment conditions. Missing values were imputed as zero to represent no change relative to untreated. Hierarchical clustering was applied using Euclidean distance and average linkage. Using the WGCNA package in R, a weighted gene co-expression network was constructed based on Pearson correlation coefficients, transformed into an adjacency matrix using an optimal soft thresholding power (β) to achieve a scale-free topology. Gene modules were identified by hierarchical clustering with the dynamic tree cut method. Each module’s expression profile was summarized by calculating the module eigengene (first principal component). Hub genes within significant modules were identified based on high connectivity and correlation with module eigengenes.

#### Statistical analysis

All statistical analyses was performed in Graphpad Prism (v9). Once compiled, datasets was tested for normality using the D’Agostino-Pearson omnibus or Shapiro-Wilk tests. Data that passed normality was analyzed using either one-way or two-way ANOVAs or non-parametric alternatives, with pos-hoc testing to determine any significant differences between groups. Datasets with only two groups were analyzed using standard t-tesst. Figure legends contain information about the number of cells analyzed, the sample size, the statistical analysis performed and the nature of the data shown on graphs (e.g., mean ± SEM). *p*-values are presented as the following; ∗ *p* < 0.05, ∗∗ *p* < 0.01, ∗∗∗ *p* < 0.005, ∗∗∗∗ *p* < 0.0001.
