# Supplementary material for: Alzheimer’s disease brain-derived tau extracts show differential processing and transcriptional effects in human astrocytes
Source: iScience. 2025 May 30;28(7):112793. doi: 10.1016/j.isci.2025.112793 (PMC12205603; doi:10.1016/j.isci.2025.112793)
Supplement: Document S1. Figure S1–S6, Tables S1 and S2, and Methods S1 [file mmc1.pdf]

iScience, Volume *28*

Supplemental information

Alzheimer's disease brain-derived tau extracts show differential processing and transcriptional effects in human astrocytes

Matthew J. Reid, Melissa Leija Salazar, Claire Troakes, Steven Lynham, Deepak P. Srivastava, Beatriz Gomez Perez-Nievas, and Wendy Noble

Supplementary Figures

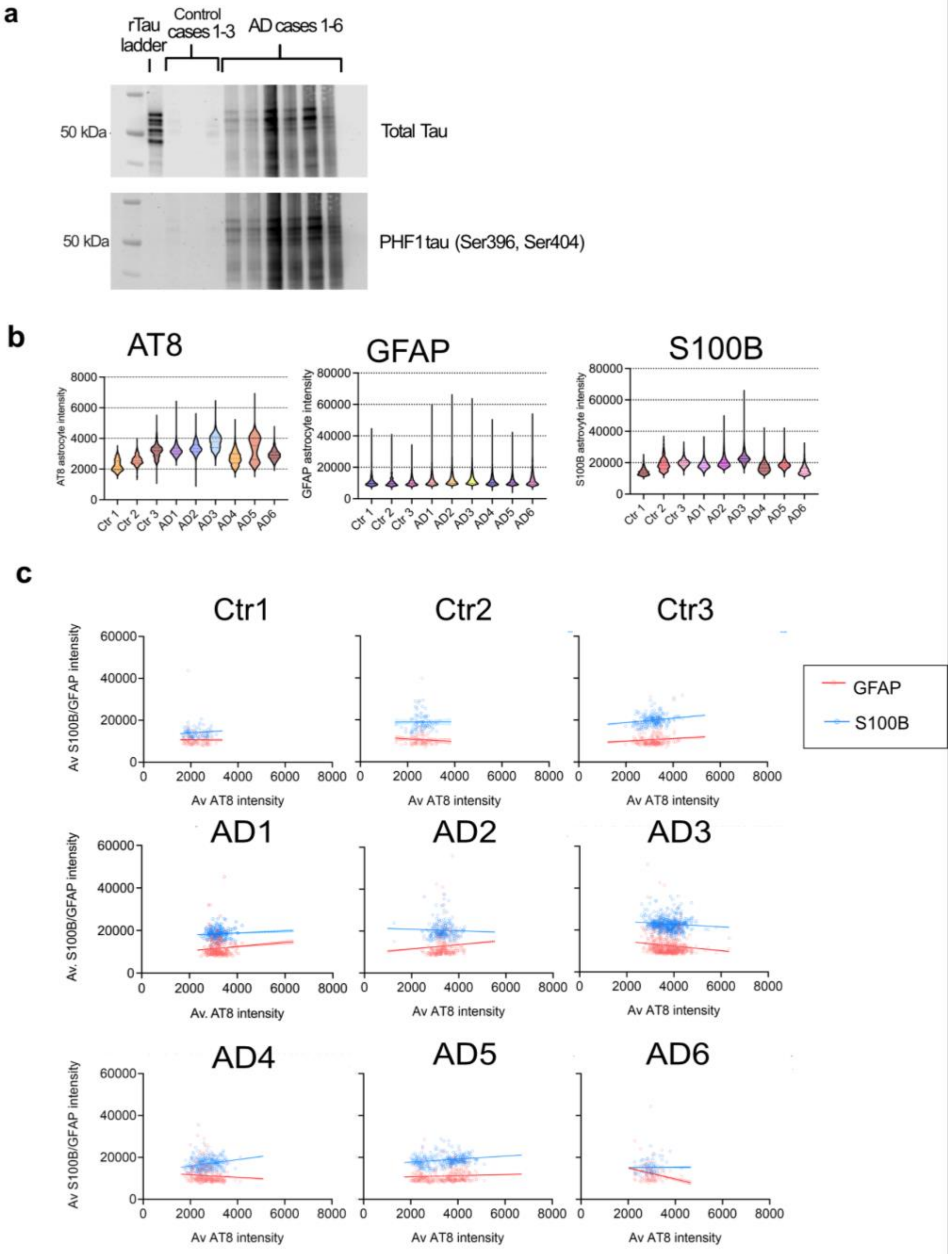

d

	Ctr1	Ctr2	Ctr3	AD1	AD2	AD3	AD4	AD5	AD6
					**S100B**				
**r**	0.1282	0.002558	0.191	0.05687	-0.03765	-0.09018	0.2348	0.2265	0.01587
**R²**	0.01644	6.54E-06	0.03647	0.003234	0.001418	0.008132	0.05513	0.05128	0.000252
**p**	0.0002	0.9621	<0.0001	<0.0001	<0.0001	<0.0001	<0.0001	<0.0001	0.4377
					**GFAP**				
**r**	-0.00702	-0.0603	0.09121	0.07605	0.06503	-0.1111	-0.07827	0.06584	-0.1776
**R²**	4.93E-05	0.003636	0.008319	0.005783	0.004229	0.01234	0.006126	0.004335	0.03153
**p**	0.837	0.2626	<0.0001	<0.0001	<0.0001	<0.0001	<0.0001	<0.0001	<0.0001

Figure S1. Characteristics of post-mortem human brain samples.

a) Sarkosyl-insoluble fractions from postmortem human control 1-3 and AD 1-6 brain, together with recombinant human tau (six main CNS isoforms), were immunoblotted using antibodies against total tau and tau phosphorylated at Ser396/404 (PHF1).
b) Violin plots showing intensity of AT8, GFAP and S100B intensity in astrocytes immunoreactive for both GFAP and S100b in individual AD (AD1-6) and control (Ctr1-3) sections. 800-2000 cells were examined per whole tissue section (n=3 ctr, n=6 AD).
c) Pearson correlation analysis of intensity of AT8 immunolabelling relative to GFAP (red) or S100b (blue) in individual astrocytes in temporal cortex for each AD and control case. 800-2000 cells were examined per whole tissue section (n=3 ctr, n=6 AD).
d) Summary statistical data from correlation analysis for each case showing r, R2 and significance of the correlation (p) between both GFAP and AT8, and S100b and AT8 in dual labelled S100b and GFAP – immunoreactive astrocytes.

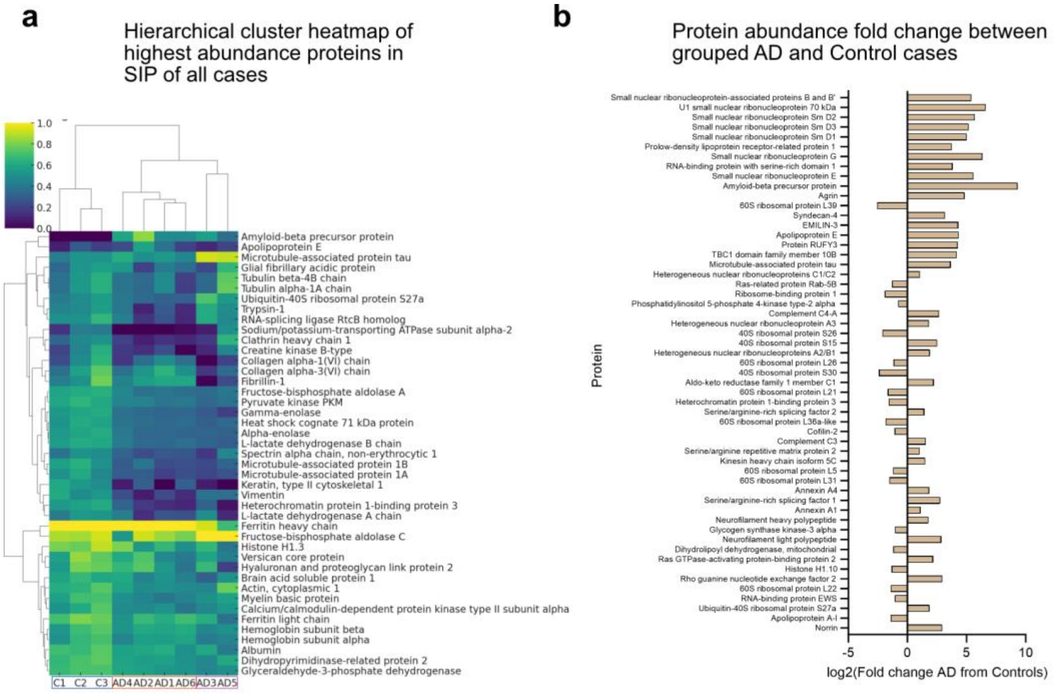

Figure S2. Characterization of sarkosyl-insoluble fractions from Alzheimer's disease and control Brains. The properties of sarkosyl-insoluble proteins isolated from AD cases (AD1-6) and control brain (C1-3) were analyzed using LC-MS/MS.

(a) The top proteins by log2 abundance in each case were combined and normalized within each sample from least abundant (dark blue) to most abundant (yellow) and are presented after an unbiased hierarchical clustering which grouped the samples into three major clusters: control samples (Ctr1-3), AD samples (AD1, AD2, AD4, AD6), and a separate AD cluster (AD3, AD5).

(b) Bar chart of the fold change of 20 most significant different proteins between AD (n=6) and control brains (n=3). Statistical significance was determined using an unpaired t-test.

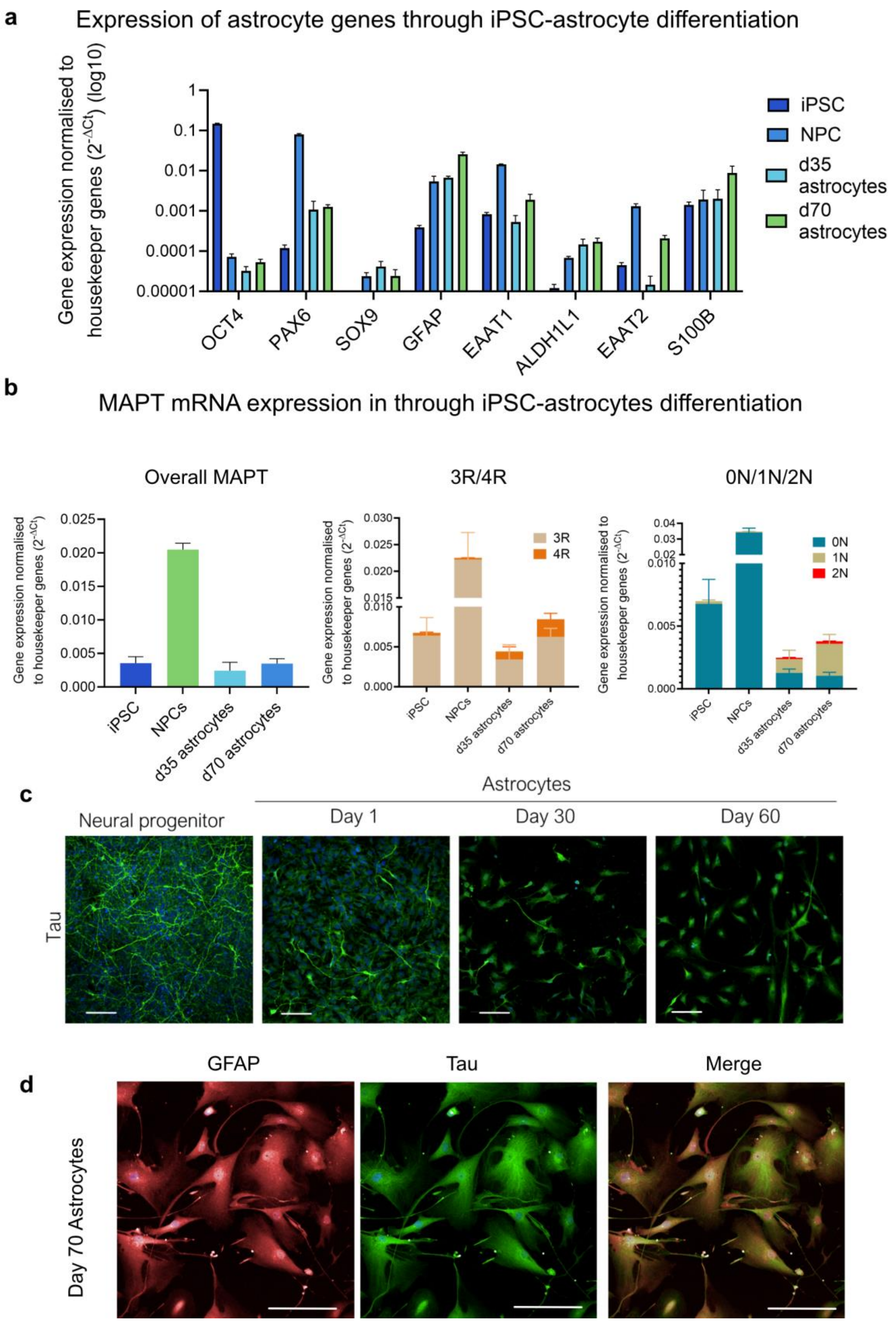

a
Expression of astrocyte genes through iPSC-astrocyte differentiation
Gene expression normalised to housekeeper genes (2^-ΔCt) (log10)
1
0.1
0.01
0.001
0.0001
0.00001
OCT4
PAX6
SOX9
GFAP
EAAT1
ALDH1L1
EAAT2
S100B
iPSC
NPC
d35 astrocytes
d70 astrocytes
b
MAPT mRNA expression in through iPSC-astrocytes differentiation
Overall MAPT
3R/4R
0N/1N/2N
Gene expression normalised to housekeeper genes (2^-ΔCt)
0.025
0.020
0.015
0.010
0.005
0.000
0.030
0.040
0.03
0.02
3R
4R
0N
1N
2N
iPSC
NPCs
d35 astrocytes
d70 astrocytes
c
Astrocytes
Neural progenitor
Day 1
Day 30
Day 60
Tau
d
GFAP
Tau
Merge
Day 70 Astrocytes

Figure S3. Characterization of human iPSC-astrocytes.

(a) Gene expression levels of mature astrocyte markers (*GFAP, EAAT1, ALDH1L1, EAAT2, S100B*), neural progenitor marker (*PAX6*) and pluripotency marker (*OCT4*) as determined by RT-qPCR in iPSC, NPC and astrocytes at day 35 or day 70 (d35, d70) of differentiation (n=3 independent differentiations).

(b) Expression of tau isoforms was analyzed in iPSC, NPC and astrocytes at differentiation days 35 and 70. RT-qPCR analysis of overall MAPT mRNA levels and expression of tau containing (4R) or missing (3R) exon 10, or containing exon 2 (1N), exons 2 and 3 (2N) or missing both exons 2 and 3 (0N) (n=3 independent differentiations).

(c) Representative immunolabelling of tau protein (green, DAKO pan-tau) using a non-isoform specific tau antibody in NPCs and astrocytes at day 1, 30 and 60 of differentiation from NPC. Scale bar = 100μm

(d) Representative immunolabelling of tau protein (green) with GFAP (red) in astrocytes differentiated for 70 days. Scale bar = 100μm

Data is from three independent differentiations of iPS-astrocytes. Data is shown as mean ± SEM. Statistical analysis was performed by one-way ANOVA with Tukey's multiple comparison to NPC stage in (a-b).

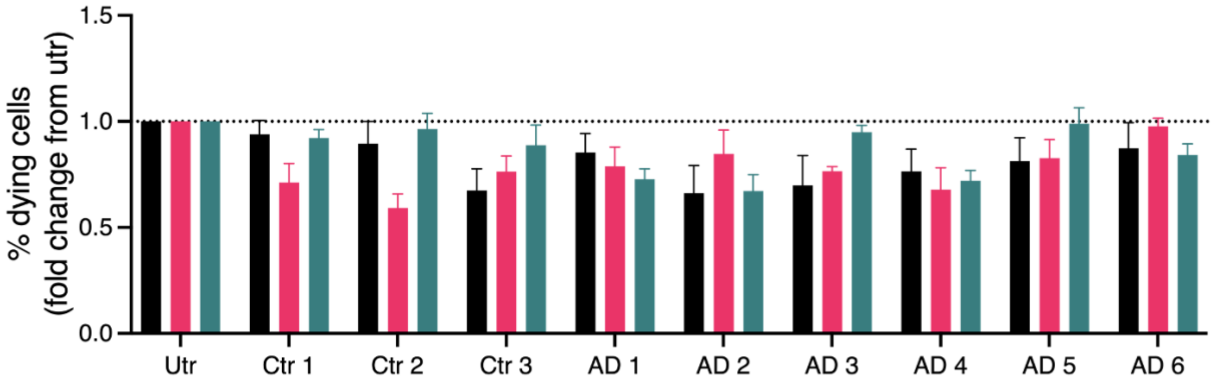

Figure S4. Analysis of nuclear morphology as an indication of cell viability.

Cell viability assessments based on "living" and "dead or dying" grouping of Hoechst 33342 labelled nuclei in astrocytes exposed to sarkosyl-insoluble tau fractions and cultured for 7 days (7d+0, black) and after tau removal from media for an additional 14 days (red) or 21 days (green). 300-500 cells were imaged per condition. N=3 Ctrl, N=6 AD. Data is from three independent differentiations of iPS-astrocytes. Data is shown as mean ± SEM. Statistical analysis was performed by one-way ANOVA with Tukey's multiple comparison.

a Astrocyte AT8 vs GFAP/S100B intensity

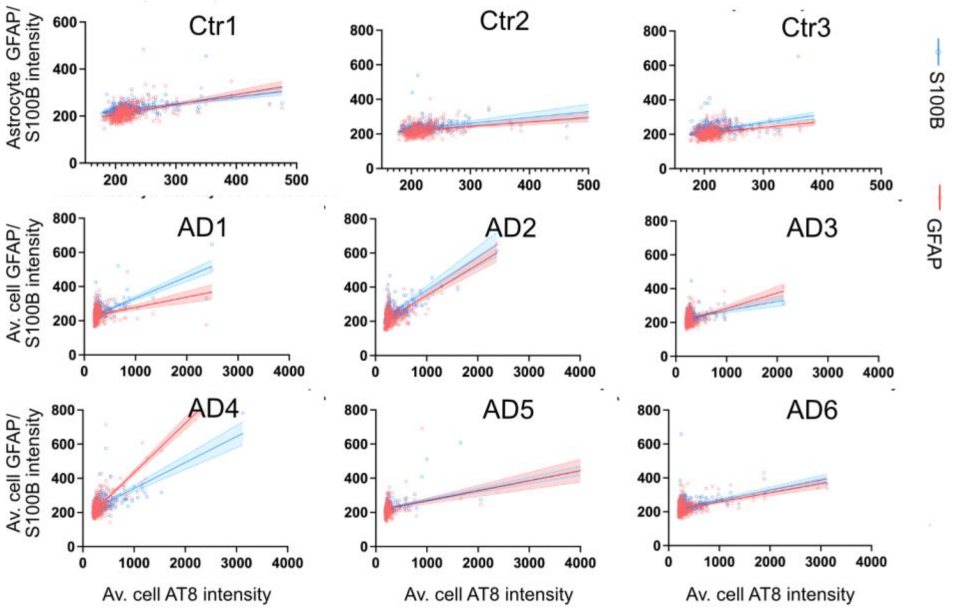

	Ctr1		Ctr2		Ctr3		AD1	
	S100B	GFAP	S100B	GFAP	S100B	GFAP	S100B	GFAP
r	0.4131	0.4377	0.2824	0.2179	0.275	0.3762	0.282	0.6174
R^2	0.1707	0.1916	0.07977	0.04748	0.07561	0.1415	0.07953	0.3812
p	<0.0001	<0.0001	<0.0001	<0.0001	<0.0001	<0.0001	<0.0001	<0.0001

	AD2		AD3		AD4		AD5		AD6	
	S100B	GFAP	S100B	GFAP	S100B	GFAP	S100B	GFAP	S100B	GFAP
r	0.5716	0.4761	0.3671	0.3207	0.7918	0.5243	0.3401	0.6626	0.4105	0.4616
R^2	0.3267	0.2267	0.1347	0.1028	0.6269	0.2749	0.1157	0.4391	0.1685	0.2131
p	<0.0001	<0.0001	<0.0001	<0.0001	<0.0001	<0.0001	<0.0001	<0.0001	<0.0001	<0.0001

b

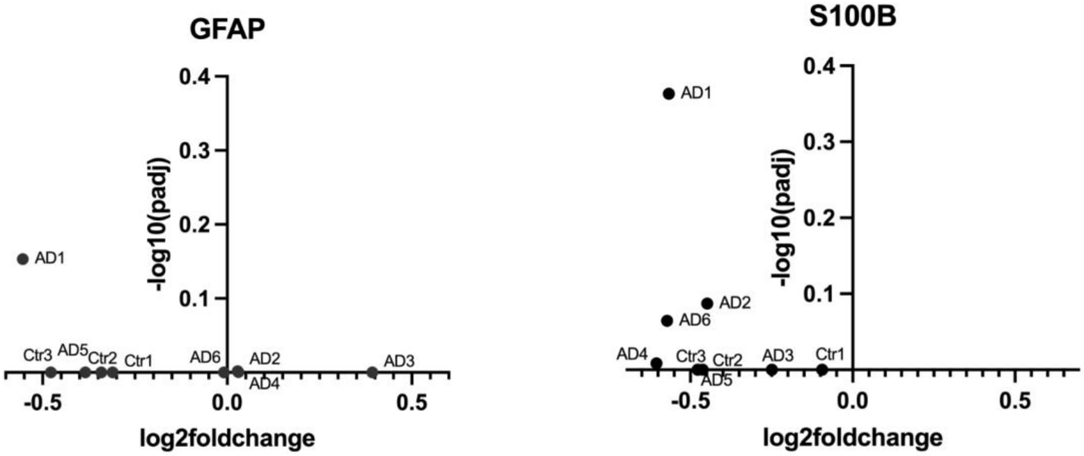

c

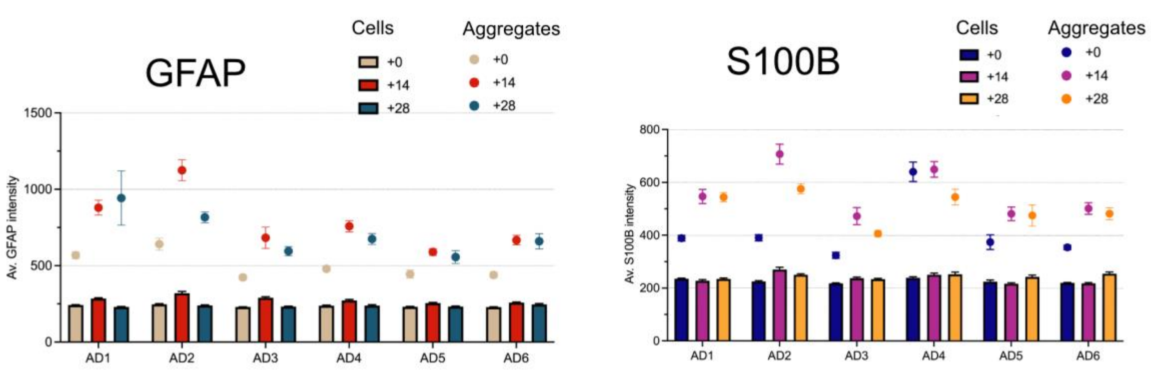

Figure S5. iPSC-astrocyte gene and protein changes following tau uptake.

(a) Scatter correlation plot of GFAP (blue) and S100B (red) with AT8 intensity of iPSC-astrocytes treated with sarkosyl-insoluble fractions from AD1-6 and Ctr1-3 brain for 7 days, and data table of resulting Pearson correlation analysis (400-500 cells were analysed across 3 experiments, N=3 ctr, N=6 AD).

(b) Gene expression fold change against -log of adjusted p-values after RNAseq of GFAP and S100B for AD (n=6) and control (n=3) treated astrocytes after 7 days relative to untreated.

(c) Average GFAP and S100B intensity in astrocytes for whole cells or in association with aggregates following exposure to sarkosyl-insoluble tau fractions for 7 days (7d+0) and after tau removal from media for 14 days (7d +14) and 28 days (7d+28) (400-500 cells were analysed across 3 experiments, N=3 ctr, N=6 AD)

Data is from three independent differentiations of iPS-astrocytes. Data is mean ± SEM. Statistical analysis in (c) by two-way ANOVA with Dunnett's multiple comparison to +0 timepoint.

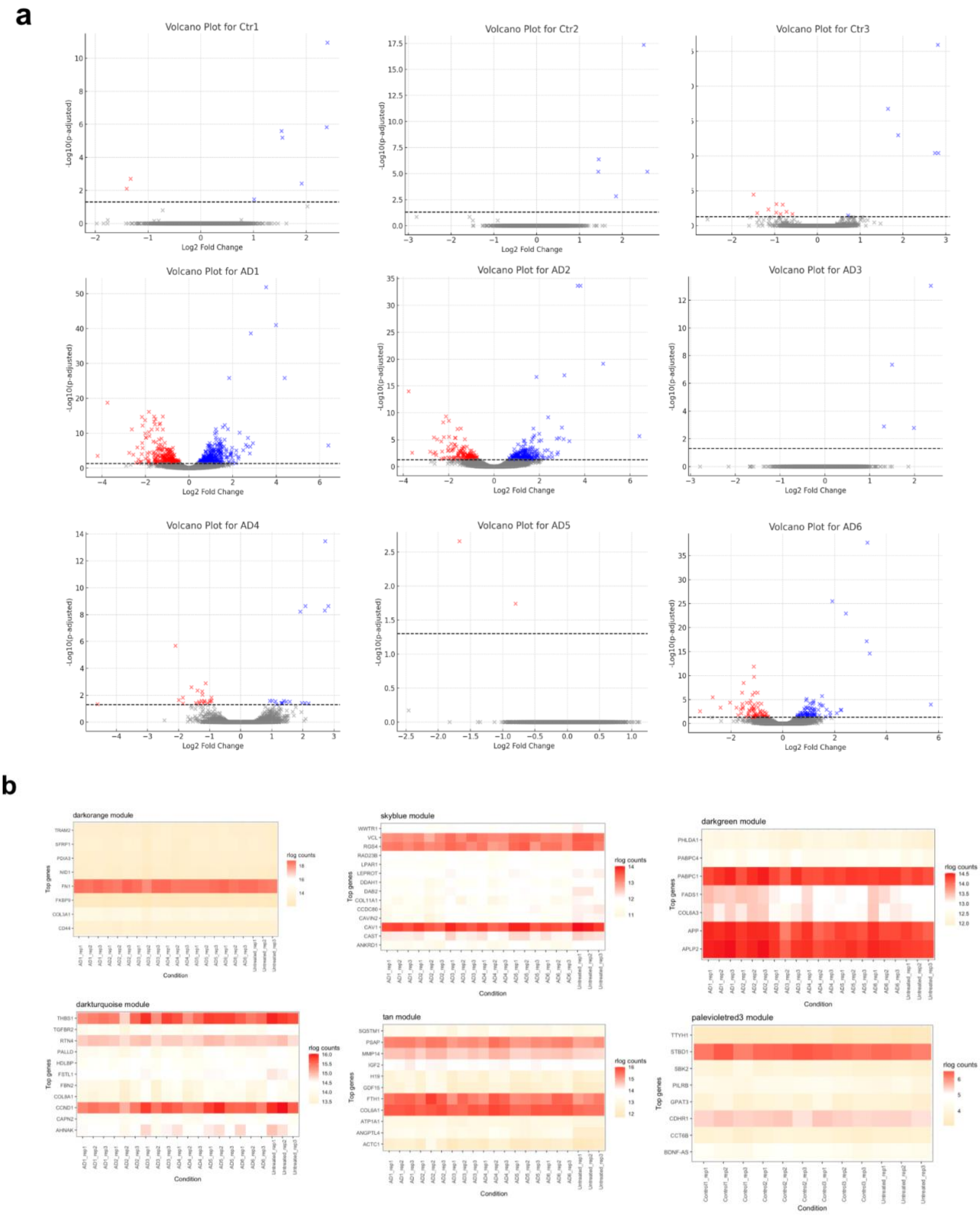
a
Volcano Plot for Ctr1
Volcano Plot for Ctr2
Volcano Plot for Ctr3
Volcano Plot for AD1
Volcano Plot for AD2
Volcano Plot for AD3
Volcano Plot for AD4
Volcano Plot for AD5
Volcano Plot for AD6
-Log10(p-adjusted)
Log2 Fold Change
b
darkorange module
skyblue module
darkgreen module
darkturquoise module
tan module
palevioletred3 module
Top genes
Condition
rlog counts

Figure S6. RNAseq analysis after astrocyte treatment with sarkosyl-insoluble tau from individual AD and control cases. iPSC-astrocytes were treated for 7 days with 35 ng/mL of tau in sarkosyl-insoluble fractions of AD cases (AD1-6) and equivalent volumes of control brains (Ctr1-3) and compared individually to untreated astrocytes after bulk RNA-sequencing.

a) Volcano plots of DEGs in astrocytes exposed to individual AD (1-6) and Control (1-3) cases vs. untreated astrocytes (significant genes, $p<0.05$, in red (upregulated)/blue(downregulated)).

b) WGCNA module heatmaps of gene expression values (in log counts) and co-expression of the highest expressed genes within each module. Data shown is 3 technical repeats for AD1-6 and Ctr1-3 and untreated astrocytes.

Supplementary Tables

Supplementary Table 1

Details of human brain samples used in this study, including age at death, sex, post-mortem delay (PMD), pathological diagnosis and ApoE status (if known)

Alias	Age	Sex	PMD	Pathological Diagnosis	ApoE g'type
AD 1	81	M	74	AD Braak VI	
AD 2	88	M	46	Alzheimer's disease: Definite Braak VI	3/4
AD 3	72	M	5	AD, Braak VI with marked amyloid angiopathy	
AD 4	97	F	12	Alzheimer's disease Braak V	2/3
AD 5	72	M	5	Alzheimer's disease Braak VI with marked amyloid angiopathy	3/3
AD 6	87	F	48	Alzheimer's disease Braak VI with moderate amyloid angiopathy	2/4
Ctr 1	77	M	11	Non-diseased	
Ctr 2	86	M	6	Non-diseased	3/3
Ctr 3	55	F	12	Minimal tau pathology consistent with HP-tau stage I	3/3

Supplementary Table 2

Primer sequences used for SYBR RT-qPCR and pre-designed TaqMan probes

**Primers for SYBR qPCR**		
**Target**	**Forward (5' – 3')**	**Reverse (5' – 3')**
0N *MAPT*	GCTGGCCTGAAAGCTGAAG	ATCGCTTCCAGTCCCGTCT
1N *MAPT*	CAACAGCGGAAGCTGAAGAA	GTGACCAGCAGCTTCGTCTT
2N *MAPT*	ACTCCAACAGCGGAAGATGT	GTGACCAGCAGCTTCGTCTT
3R *MAPT*	AGGCGGGAAGGTGCAAATA	GCCACCTCCTGGTTTATGATG
4R *MAPT*	CGGGAAGGTGCAGATAATTAA	TATTTGCACACTGCCGCCT
ALDH1L1	CCAAAGTCCTGGAGGTTGAA	TAACTCCAGGCCATCACACA
B-ACTIN	TCGTGCGTGACATTAAGGAG	AGGAAGGAAGGCTGGAAGAG
EAAT1	TCAAGTTCTGCCACCCTACC	AATGAAAATGGCAGCCAAAG
EAAT2	TCAGTCAATGTTGTGGGTGA	GTTGCTTTCCCTGTGGTTCT
GAPDH	AGCCTCAAGATCATCAGCAA	CTGTGGTCATGAGTCCTTCC
GFAP	GAGTCCCTGGAGAGGCAGAT	GTAGGTGGCGATCTCGATGT
MAP2AB	AAACTGCTCTTCCGCTCAGACACC	GTTCACTTGGGCAGGTCTCCACAA
MAPT	GTCGAAGATTGGGTCCCT	GACACCACTGGCGACTTGTA
OCT4	TTGGGCTCGAGAAGGATGTG	GTGAAGTGAGGGCTCCCATA
PAX6	GCCAGAGCCAGCATGCAGAACA	CCTGCAGAATTCGGGAAATGTCG

S100β	AAAGAGCAGGAGGTTGTGGA	CGTGGCAGGCAGTAGTAACC
SOX9	AGGTGCTCAAAGGCTACGAC	GCTTCTCGCTCTCGTTCAGA
SERPIN3A	CGTGGTGGAGCTGAAGTACA	GCCCAGCTGGAGAAGTATGT
STAT3	GGCATTCGGGAAGTATTGTCG	GGTAGGCGCCTCAGTCGTATC
TFEB	CCAGAAGCGAGAGCTCACAGAT	TGTGATTGTCTTTCTTCTGCCG
TaqMan Probes		
Target	Product ID	Fluorescence
B-ACTIN	Hs01060665_g1	VIC-MGB_PL
GAPDH	Hs02786624_g1	VIC-MGB_PL
MAPT (total)	Hs00902193_m1	FAM-MGB
0N *MAPT*	Hs00902188_m1	FAM-MGB
1N *MAPT*	Hs00902978_m1	FAM-MGB
2N *MAPT*	Hs00902314_m1	FAM-MGB
3R *MAPT*	Hs00902192_m1	FAM-MGB
4R *MAPT*	Hs00902312_m1	FAM-MGB

Methods S1

Supplementary Code

- The following python script was used for hierarchical clustering and heatmap visualization of binary-transformed phosphorylation data from tau PTM analysis.

```
import pandas as pd
import seaborn as sns
import matplotlib.pyplot as plt

# Load data
file_path = 'your_data_file.xlsx' # Replace with your data file path
data = pd.read_excel(file_path, index_col=0)

# Convert data to binary
binary_data = data.applymap(lambda x: 1 if x > 0 else 0)

# Rename columns if needed
binary_data.rename(columns={'C3': 'Ctr3'}, inplace=True)

# Perform hierarchical clustering
clustermap = sns.clustermap(binary_data, method='ward', metric='euclidean', cmap='Oranges',
figsize=(15, 12),
                        cbar=False, linewidths=.5, linecolor='black')

# Adjust row labels
clustermap.ax_heatmap.set_yticks([x + 0.5 for x in range(len(binary_data.index))])
clustermap.ax_heatmap.set_yticklabels(binary_data.index)
clustermap.ax_heatmap.tick_params(axis='y', which='major', labelsize=8, labelrotation=0)

# Save the heatmap as an image
heatmap_image_path = 'clustered_binary_phosphorylation_heatmap.png'
clustermap.savefig(heatmap_image_path)

# Export clustered data
ordered_data = binary_data.iloc[clustermap.dendrogram_row.reordered_ind,
clustermap.dendrogram_col.reordered_ind]
export_path = 'clustered_binary_phosphorylation_data.xlsx'
ordered_data.to_excel(export_path)
```

- The following script was used for hierarchical clustering and heatmap visualisation of differentially expressed genes (DEGs) across multiple treatment conditions, using log2 fold change value.

```
import pandas as pd
```

```
import numpy as np
import seaborn as sns
import matplotlib.pyplot as plt
from scipy.cluster.hierarchy import linkage
from scipy.spatial.distance import pdist

# Load log2 fold change data from multiple conditions
# Assumes you have CSVs with gene identifiers as rows, one column per condition
# Replace 'deg_data.csv' with your actual filename
df = pd.read_csv('deg_data.csv', index_col=0)

# Replace missing values (non-significant genes) with 0 (no change)
df_filled = df.fillna(0)

# Optional: filter out genes with all zeros (no change in any condition)
df_filtered = df_filled.loc[~(df_filled == 0).all(axis=1)]

# Clustering using Euclidean distance and average linkage
row_linkage = linkage(pdist(df_filtered, metric='euclidean'), method='average')
col_linkage = linkage(pdist(df_filtered.T, metric='euclidean'), method='average')

# Plot heatmap
sns.clustermap(
    df_filtered,
    cmap='RdBu_r',
    center=0,
    row_linkage=row_linkage,
    col_linkage=col_linkage,
    figsize=(10, 10),
    xticklabels=True,
    yticklabels=False
)

plt.savefig("DEG_heatmap.png", dpi=300, bbox_inches='tight')
plt.close()

print("Heatmap saved as DEG_heatmap.png")
```
